# Accessing Arenes via the Hydrodeoxygenation of Phenolic Derivatives Enabled by Hydrazine

**DOI:** 10.1021/acscatal.4c06061

**Published:** 2025-02-10

**Authors:** Benedetta Di Erasmo, Inna Perepichka, Hui Su, Luigi Vaccaro, Chao-Jun Li

**Affiliations:** † Laboratory of Green S.O.C. − Dipartimento di Chimica, Biologia e Biotecnologie, Università Degli Studi di Perugia, Via Elce di Sotto 8, Perugia 06123, Italy; ‡ Department of Chemistry, and FRQNT Centre for Green Chemistry and Catalysis, McGill University, 801 Sherbrooke Street West, Montreal, Quebec H3A 0B8, Canada

**Keywords:** hydrodeoxygenation, phenols, heterogeneous catalyst, hydrazone, arenes, palladium

## Abstract

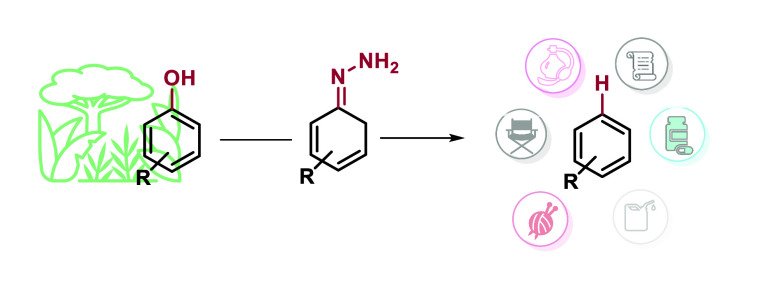

Hydrodeoxygenation (HDO) is an effective method for converting lignin and its derived phenolic compounds to value-added aromatic chemicals and fuels. Efforts to exploit molecular hydrogen have been made to remove the hydroxyl group in lignin-derived phenolic compounds to make them appealing for the chemical industry. However, these processes rely on high pressure and expensive catalysts, presenting challenges in terms of safety, hydrogen storage, and cost-effectiveness. This highlights the demand for alternatives under more accessible reaction conditions. Herein, we present a methodology for the HDO of phenols and naphthols using Pd/C as a commercial heterogeneous catalyst employing hydrazine as a dual reagent for reducing and hydrazone formation. This paper presents an applicable substrate scope for the HDO of different naphthols and phenols including pharmaceutically relevant molecules such as paracetamol. Additionally, highly challenging steroid derivatives, such as β-estradiol, have been hydrodeoxygenated.

## Introduction

**Scheme 1:**
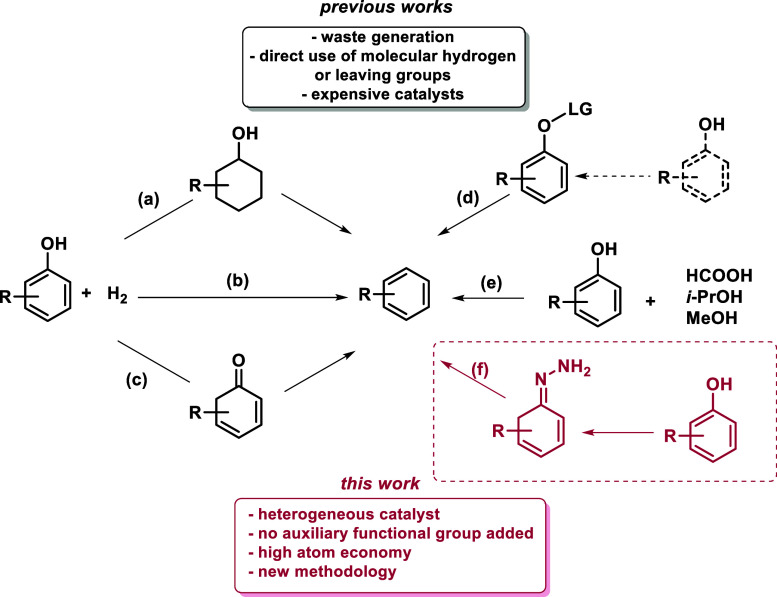
Possible Pathways for the Hydrodeoxygenation of Phenols to Arene Derivatives

Arenes are key chemical intermediates as they provide access to a broad range of fine chemicals such as fuels, textiles, fragrances, pharmaceuticals, paper products, and rubbers.^1−6^ They are mostly produced from fossil fuels, mainly through the catalytic reforming of naphtha in petroleum refineries via intense energy demanding processes with a large production of waste.^7,8^ To overcome this environmental problem, a strategy that can be implemented to obtain arenes in a sustainable manner is starting from renewable sources. In this sense, potentially using lignin-derived phenols is a valid alternative.^9^ One of the main methods by which arene derivatives are produced from phenols is known as hydrodeoxygenation (HDO).^10−16^ The most common pathways require elevated pressures of molecular hydrogen as it is the most atom-efficient and economical reductant available. For example, a possible route (Scheme 1a) consists in a first direct ring-saturation followed by a dehydration process to remove the hydroxyl group and a dehydrogenation step to form the benzene derivative.^17^ The easiest pathway would be the direct C–O cleavage to access the benzene (Scheme 1b), but this one is also less favored due to the high energy barrier to break the C–O bond.^18−20^ Another possible mechanism pursues a tautomerization of the phenol to the correspondent keto form followed by hydrogenation and dehydration to yield benzene (Scheme 1c).^21−25^ Despite the great usefulness of these methods, they are energy demanding: high temperatures and elevated pressures of molecular hydrogen are required, and the high fugacity of H_2_ poses significant challenges, particularly in closed environments. Some efforts in using low temperatures or low pressures have been pursued with transition metals supported onto carbonaceous materials,^26,27^ TiO_2_,^28−30^ Nb_2_O_5_,^31,32^ and ZrO_2_,^33^ and homogeneous iridium catalysts.^20^ However, further work is needed to properly address the problem and broaden the substrate applicability. This addition ties the previous efforts to the need for alternative approaches and helps to clarify the potential value of exploring new methods. For this reason, finding alternative routes to promote HDO without the use of molecular hydrogen may be useful in certain cases. In the literature, there are some examples where an auxiliary functional group is introduced on the oxygen of the phenolic unit to form a better leaving group to access the arene derivative after the group removal (Scheme 1d). Groups that have been used for this purpose are hydro silanes,^34^ B_2_Pin_2_,^35^ 2,4,6-trichloro-1,3,5-triazine (TCT),^36^ and triflates.^37−40^ Another type of HDO without employing molecular hydrogen is the catalytic transfer hydrogenation/hydrogenolysis (CTH), which uses liquid organic hydrogen carriers (LOHCs) instead of molecular H_2_ to transfer hydrides (Scheme 1e).^14,41−43^

Recently, our group developed a strategy where hydrazones can serve as organometallic equivalents (HOME) undergoing various reactions with carbon electrophiles, such as addition or cross-coupling reactions.^44,45^ This chemistry has highly sustainable features since it gives efficient transformations under mild reaction conditions, generating only nitrogen gas and water as inert byproducts. The idea behind HOME-chemistry is that carbonyl groups can be converted into methylene derivatives, as in the Wolff–Kishner reduction.^46,47^ Applying this concept to our purpose of accessing arenes from phenols, we have exploited the Pd-catalyzed selective reduction of phenols (Scheme 1f) to cyclohexanones and the fact that the latter couple with hydrazine to form hydrazones. Mechanistic insights revealed that once hydrazone is formed, it is converted to the corresponding arylamine, thanks to a rearomatization process together with a N–N cleavage promoted by the Pd action. Finally, a C–N cleavage gives the desired HDO product. Herein, we present the current state of exploration of this novel methodology, including its conceptualization, optimization of reaction conditions, and application to various substrates. This reaction can serve as a starting point for other applications involving more complex molecules (e.g., β-estradiol) where the use of molecular hydrogen is limited.

## Results and Discussion

To identify the best conditions for HDO of hydroxyarenes using hydrazone chemistry, we used the reaction between 1-naphthol and hydrazine monohydrate as a model. We hypothesized that hydrazine could serve dual roles: as a reductant to convert 1-naphthol to α-tetralone and as a coupling agent to form the corresponding hydrazone from α-tetralone. To facilitate the first step of the reaction, a metal catalyst is required as a “hydride-shuttle” from hydrazine to 1-naphthol.^48,49^ We tested various catalysts, including homogeneous and heterogeneous forms of Ni, Ru, Rh, and Pd in the presence of different bases. The Pd/C catalyst was the most effective, achieving the highest conversion of 1-naphthol (Tables S1 and S2) to naphthylamine instead of naphthalene, **2a**. This indicated that under the conditions employed, the reaction favored the reductive N–N bond cleavage over the expected C–N bond cleavage. To promote C–N bond cleavage, trifluoroacetic acid (TFA) was used as it is often employed in the removal of nitrogen-containing protecting groups.^50^ These conditions yielded a first 52% NMR yield of naphthalene **2a** (entry 1, Table 1). The presence of water is detrimental for the reaction since we observed a consistent drop in the conversion (88% vs 37%; entries 1–2, Table 1). TFA can, in fact, undergo hydrolysis in the presence of water, losing its activity. Also, dilution is disadvantageous for our reaction (entries 1–3, Table S2). To investigate whether the different acidities of other additives could further help the reaction, we conducted a screening with various organic and inorganic acids (entries 3–7, Table 1). Even though the conversion of starting material **1a** is high in the absence of added water in all tests, more side products are formed when acids other than TFA are used. We then focused on optimizing the Pd loading (entries 8–11, Table 1): through our experiments, we determined that using 15 mol % Pd/C provided the best results. Temperature screening was conducted too, finding that high temperature is necessary for excellent conversion and improved selectivity (entries 10–11). Different hydrazine sources were tested too, such as hydrazine in THF, hydrazine acetate, and dimethyl hydrazine (entries 12–14, Table 1), proving that N_2_H_4_ in THF is the best solution, leading to 60% of naphthalene **2a**. Since the major byproduct in this control reaction is the reduced form of naphthalene (1,2,3,4-tetrahydronaphthalene), we explored whether the reaction could proceed through the formation of an oxime derivative under less harsh reductive conditions. To test this, we attempted to remove hydrazine and use oximes, specifically NH_2_OH·HCl and NHOHBoc (entries 15–16, Table 1). However, these conditions did not result in any conversion. Furthermore, the reaction is highly air-sensitive and requires an argon atmosphere (entry 17). A blank test (entry 18) produced a hydrazone dimer. Further experiments (Scheme S2) confirmed that this is a side product of the reaction. Finally, we did a test with molecular hydrogen (entry 20): as expected, these conditions provide a reductive environment that is too harsh, and the only product formed is the completely hydrogenated form, tetralin **3a** (95% NMR yield).

**Table 1:**
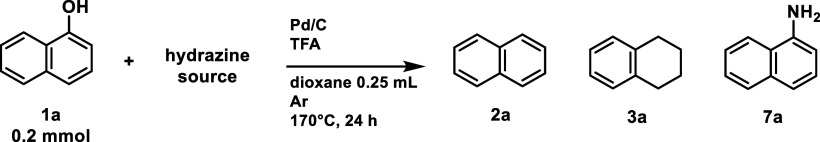
Acid, Hydrazine, and Pd/C Optimizations^a^

entry	Pd/C (mol%)	additive	hydrazine source	**1a** (%)^b^	**2a** (%)^b^	**3a** (%)^b^	**7a** (%)^b^
1	7	TFA	N_2_H_4_·H_2_O	22	52	3	35
2	7	TFA^c^	N_2_H_4_·H_2_O	63	23	14	0
3	7	CH_3_COOH	N_2_H_4_·H_2_O	18	33	32	17
4	7	CF_3_SO_3_H	N_2_H_4_·H_2_O	16	5	22	57
5	7	B(OH)_3_	N_2_H_4_·H_2_O	20	12	5	63
6	7	KPF_6_	N_2_H_4_·H_2_O	35	21	17	27
7	7	HBF_4_ (48 wt % water)	N_2_H_4_·H_2_O	100			
8	10	TFA	N_2_H_4_·H_2_O	20	54	6	20
9	15	TFA	N_2_H_4_·H_2_O	11	57	16	16
10^d^	15	TFA	N_2_H_4_·H_2_O	27	35	23	29
11	20	TFA	N_2_H_4_·H_2_O	4	57	25	14
12	15	TFA	N_2_H_4_ in THF [1.0 M]	3	60	34	3
13	15	TFA	NH_2_NH_3_^+^	100			
			CH_3_COO^–^				
14	15	TFA	NH_2_N(CH_3_)_2_	decomposition of the starting material			
15	15	TFA	NHOHBoc	100			
16	15	TFA	NH_2_OH·HCl	100			
17^e^	15	TFA	N_2_H_4_ in THF [1.0 M]	100			
18	0	TFA	N_2_H_4_ in THF [1.0 M]	only hydrazone dimer has been formed			
19	0		N_2_H_4_ in THF [1.0 M]	60^f^	8	5	4
20	15	TFA	g	5		95	

a Reaction conditions: **1a** (0.2 mmol), hydrazine source (3 equiv.), Pd/C, acid additive (1 equiv.), dioxane (0.25 mL), 24 h, 170 °C, Ar, 10 mL pressure tube.

b Obtained using CH_2_Br_2_ as the external standard.

c 20 μL of H_2_O is added.

d 140 °C.

e In air.

f The remaining 23% is 1,2,3,4-tetrahydroxy-1-naphthol.

g H_2_ employed instead of hydrazine.

After discovering that TFA is crucial in the reaction system, we tested different ratios of hydrazine to TFA to maximize the yield of naphthalene **2a** (Table 2). We observed that when the hydrazine to TFA ratio is less than 1 (entries 1–3), **2a** is produced only in trace amounts, with unreacted 1-naphthol **1a** dominating. At a 1:1 ratio (entry 4), the yield of **2a** improved to 51%. Increasing the hydrazine to 1.5 equiv. while keeping TFA at 1.0 equiv. (hydrazine/TFA ratio = 1.5) slightly boosts the yield to 55% (entry 5). A further increase to 2.0 equiv. of hydrazine (entry 6) significantly raises the yield of **2a** to 70%. However, when we tested 3.0 equiv. of hydrazine and 1.0 equiv. of TFA (entry 7), the yield of **2a** dropped to 60% because of the formation of hydrogenation byproducts. By comparing entry 2, entry 5, and entry 6, we can conclude that the optimal hydrazine to TFA ratio is 2. Ratios lower than this result in poor conversion, while higher ratios produce unwanted hydrogenation byproducts. We conclude that 2 equiv. of hydrazine are necessary to both reduce 1-naphthol and form the corresponding hydrazone, while just 1 equiv. of TFA is sufficient for the C–N cleavage to occur. Finally, analysis of time dependence showed that 24 h is optimal for conversion (Table S3).

**Table 2:**
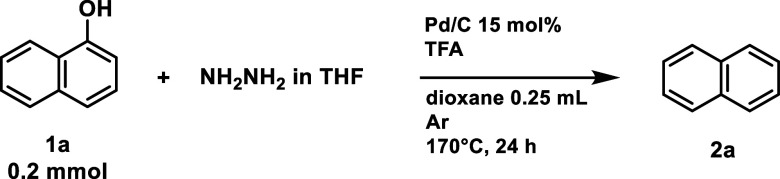
Hydrazine and TFA Amount Screening^a^

entry	N_2_H_4_ in THF (equiv.)	TFA (equiv.)	Ratio N_2_H_4_/TFA (equiv./equiv.)	NMR yield of **2a**^b^ (%)
1	0	1.0	0	3
2	1.0	2.0	0.5	12
3	2.0	3.0	0.7	5
4	1.0	1.0	1	51
5	1.5	1.0	1.5	55
6	2.0	1.0	2	70
7	3.0	1.0	3	60

a Reaction conditions: **1a** (0.2 mmol), N_2_H_4_ in THF [1.0 M], Pd/C (15 mol %), TFA, dioxane (0.25 mL), 24 h, 170 °C, Ar, 10 mL pressure tube.

b Obtained using CH_2_Br_2_ as the external standard.

The applicability of the optimized process has been explored by varying the phenol substrate (Table 3). The reaction proceeded satisfactorily with 1-naphthol and alkylated 1-naphthols (**1a**–**c**), in up to a 70% yield. We observed that the methoxy group tolerates the reaction conditions (**2d**), while the halogen-containing (**1e**) and carboxyl-containing groups (**1f**) lead to considerable defunctionalization, yielding naphthalene **2a**. This new HDO method also tolerates heterocycles, such as carbazole **2g**, which is obtained from 4-hydroxycarbazole **1g** with a moderate yield of 40%. Aniline **2h** is produced with yields of up to 30% from both 4-aminophenol **1h** and 3-aminophenol **1i**. The chemoselectivity for these substrates is lower than that for naphthols because, in addition to the main side product (hydrogenation of the aromatic ring), a deaminated side product is also formed. This can be explained by the proposed mechanism (Scheme 4c), where the amino group undergoes C–N bond cleavage facilitated by TFA. The higher yield from 4-aminophenol may possibly be due to the concurrent deactivation of 3-aminophenol. Ethylbenzene serves as a key intermediate to obtain styrene and its subsequent product polystyrene, as well as a solvent for paints and a fuel additive in gasoline.^51,52^ As for the majority of arene derivatives, this intermediate is produced from fossil fuels. However, with this new method, we were able to obtain up to 42% of isolated yield from both 2-ethylphenol **1j** and 4-ethylphenol **1k** (models of lignin-derived phenols). Simple biphenyl **2l** was obtained in a very good yield (56%), with a small percentage of the corresponding dearomatized product (13%).

**Table 3:**
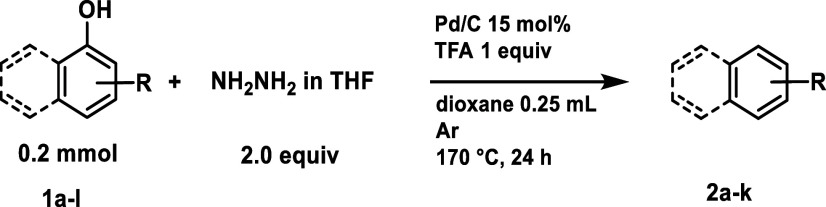

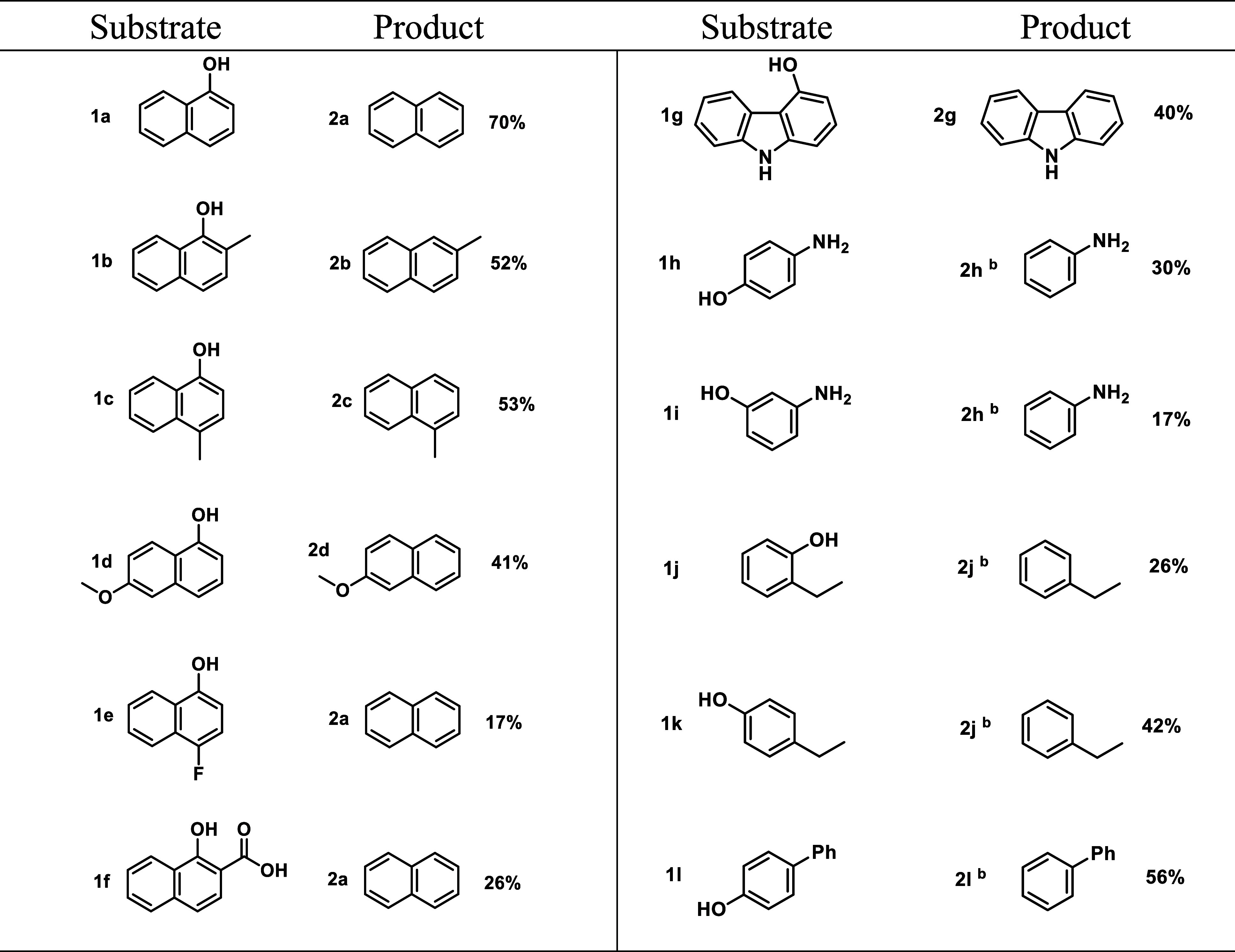
Hydrodeoxygenation of Different 1-Naphthols and Phenols via Hydrazone Chemistry^a^

a Reaction conditions: **1a**–**l** (0.2 mmol), N_2_H_4_ in THF (1.0 M, 2.0 equiv.), Pd/C (15 mol %), TFA (1.0 equiv.), dioxane (0.25 mL), 24 h, 170 °C, Ar, 10 mL pressure tube. Isolated yields are shown.

b N_2_H_4_·H_2_O (3.0 equiv).

**Scheme 2:**
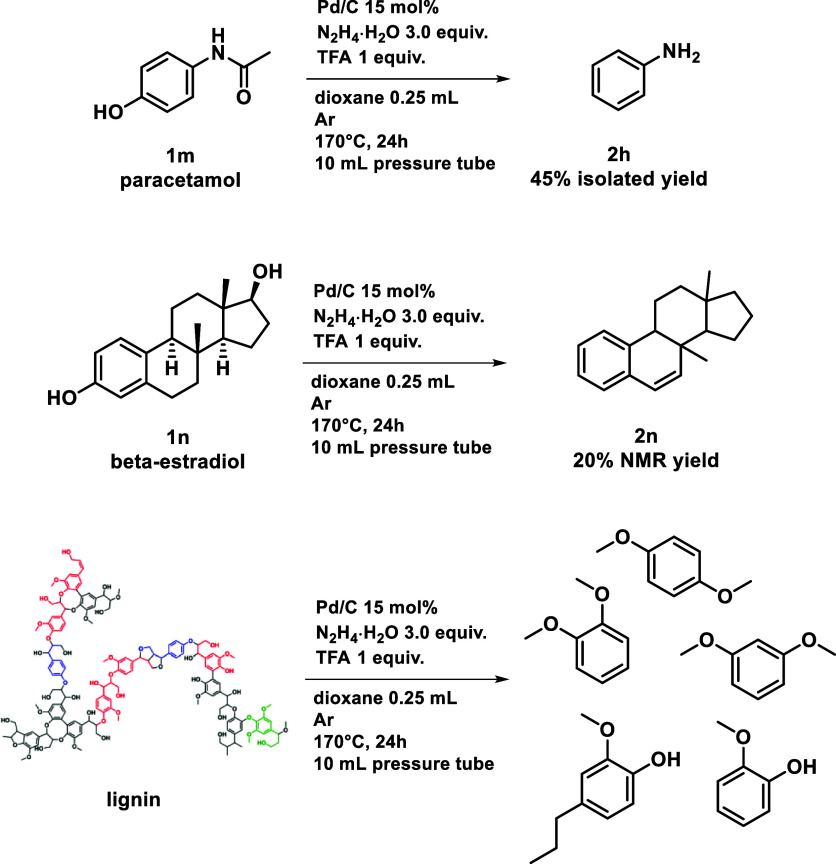
Hydrodeoxygenation on Pharmaceutically Relevant Phenols and on Lignin

Pharmaceutically relevant phenols have been tested (Scheme 2). From commercial paracetamol **1m**, we obtained aniline **2h** in a reasonable amount (45%). This indicates that our method can be a viable alternative to the decommissioning of paracetamol, embracing a circular economy approach to address the growing challenge of the disposing of expired APIs.^53^ β-estradiol is an important human sex hormone in both women and men:^54^ for the first time, this highly challenging steroid derivative have been hydrodeoxygenated to a phenanthrene derivative. Finally, to test a broader applicability of this method, we carried out the reaction with pure lignin. By GC/MS, we detected dimethoxybenzene derivatives guaiacol and 4-propyl-2-methoxy-phenol. Isolation of the target products was not achieved, likely due to the formation of several side products including phenolic ester dimers. Nonetheless, these findings represent a valuable starting point for future investigations and applications.

**Scheme 3:**
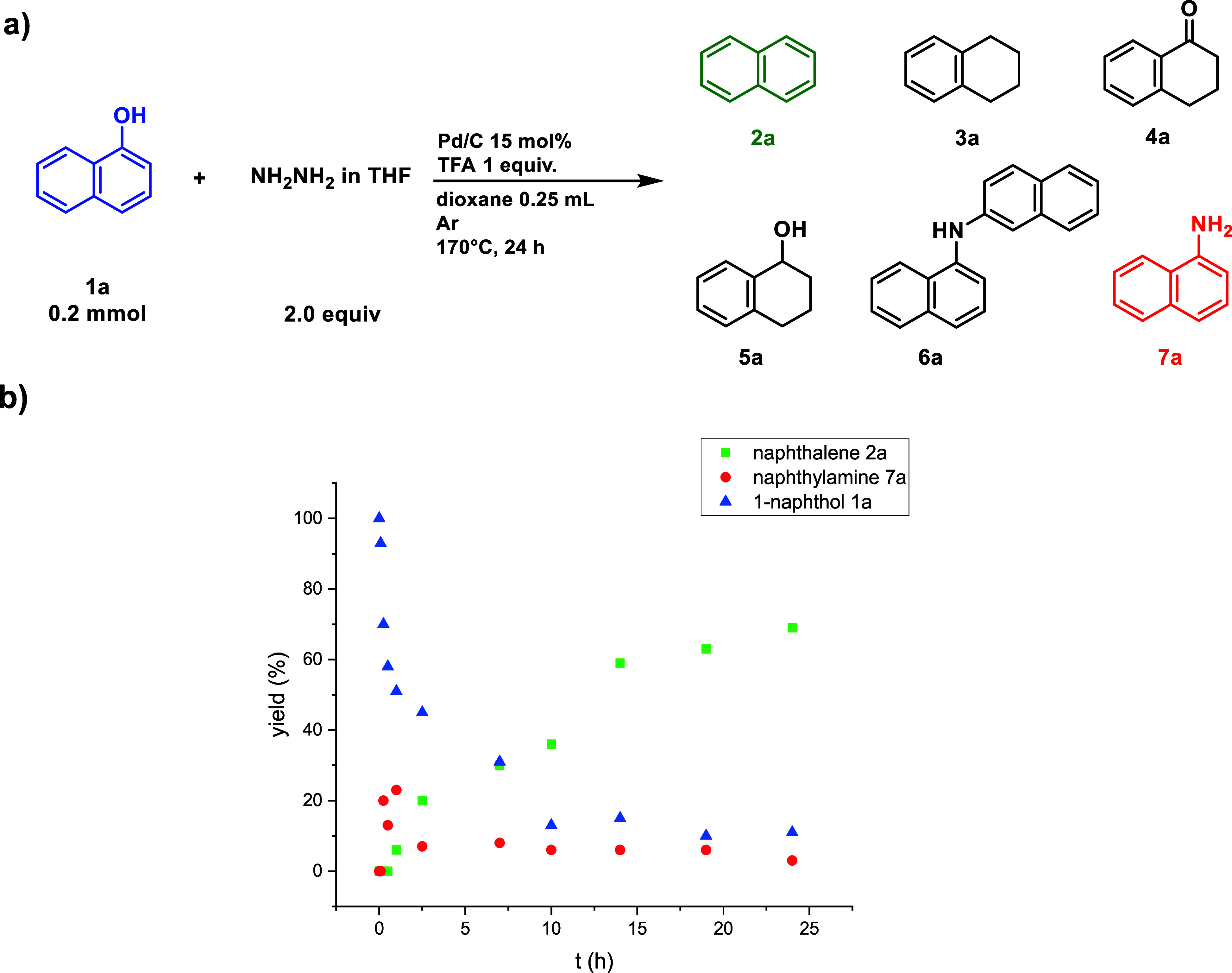
(a) Intermediates and Byproducts Isolated during the Optimization Processes. (b) Kinetic Studies of 1-Naphthol

To gain preliminary insights into the mechanism, we pursued kinetic studies (Scheme 3). It became evident that during the first few hours, the decrease in starting material and the increase in product yield did not correlate as expected. In the case of 1-naphthol **1a**, we observed that its kinetic profile can be divided into two distinct trends: for the initial one, up to 5 h, the concentration of 1-naphthol follows a first-order kinetic model (Figure S1) with *k* = 8.3 × 10^–4^ M^–1^. Beyond this point, the reaction rate reaches a plateau, likely due to saturation of all accessible active sites of the heterogeneous catalyst. On the other hand, naphthalene starts to be formed after 1 h of reaction time with a linear dependence with respect to time. This investigation helped us to shape the fact that between 1-naphthol and naphthalene, something else should be formed. Specifically, the isolation of various reaction products allowed us to establish that naphthylamine **7a** is a crucial intermediate in the conversion of 1-naphthol **1a** to naphthalene **2a**.

**Scheme 4:**
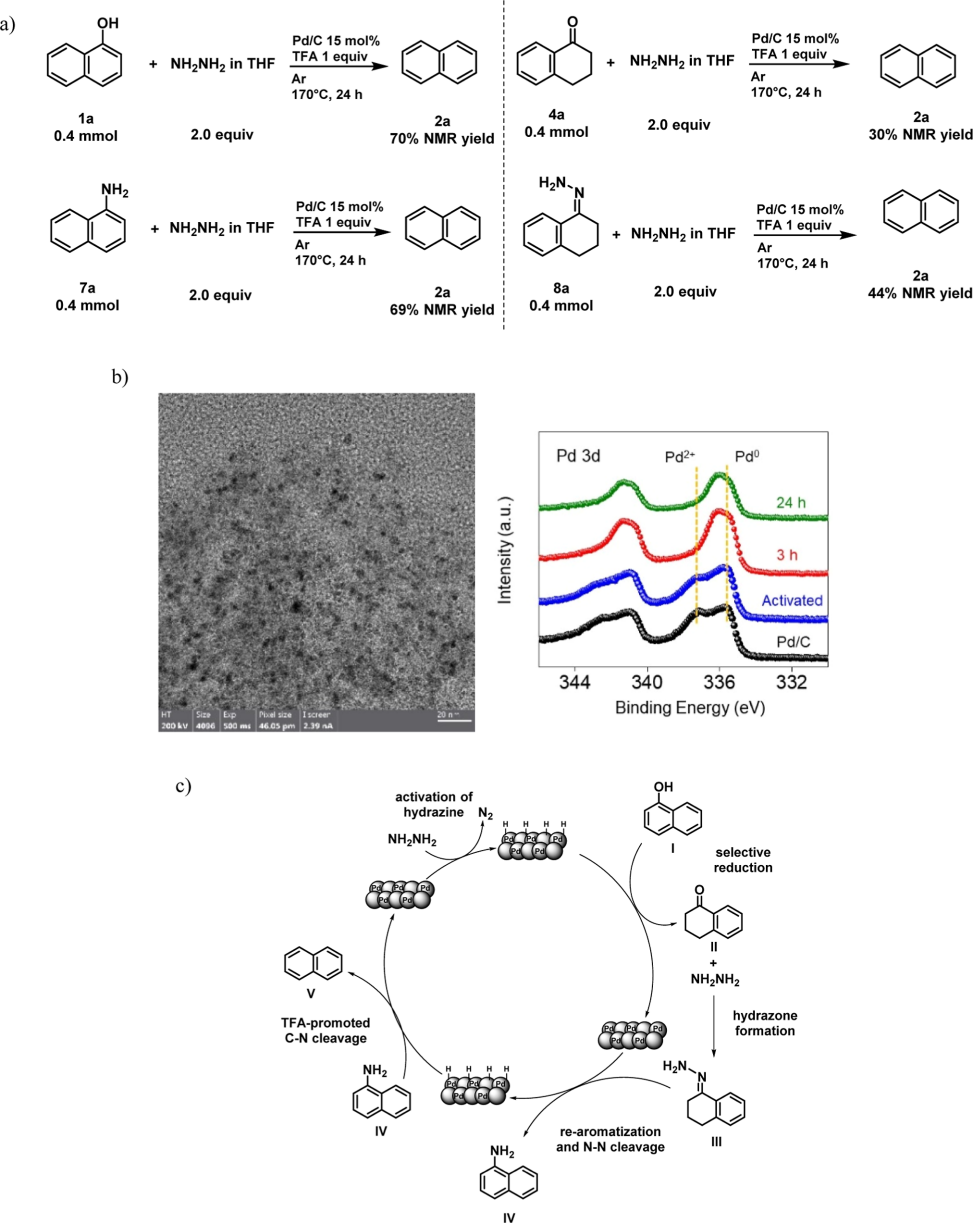
(a) Mechanistic Controls; (b) Typical Characterization Results of Used Catalyst Include the TEM Image (Left) of the Catalyst after a 24 h Reaction and the Pd 3d XPS Analyses (Right) of the Catalyst at Various Stages of the Reaction; and (c) Proposed Mechanism for the HDO of Phenols to Arenes

To further demonstrate that naphthylamine is an intermediate of the reaction, we conducted control experiments (Schemes 4a and S2) using various intermediates observed in previous tests. First, we observed that starting from naphthylamine **6a**, we obtained a 69% yield of naphthalene **2a**, confirming that naphthylamine **6a** is an intermediate in the reaction. Next, we performed the reaction starting from α-tetralone and the corresponding hydrazone to investigate if they could be potential intermediates in the reaction, as suggested in a previous paper on the formation of aniline.^55^ With both species, we were able to obtain naphthalene in good yields (30% and 44%). To expand the investigation of the mechanism, we performed structural characterization on the catalysts at different stages of the reaction: before and after the activation, after 3 and 24 h (Scheme 4b and Supporting Information). The scanning electron microscopy (SEM) and transition electron microscopy (TEM) images of the used catalyst indicate that the Pd/C catalyst’s support morphology and the size of Pd nanoparticles remain unchanged at the end of the reaction. This suggests that the catalyst remains potentially active after the first run. Indeed, we performed a recycle test with recovering Pd/C and still obtained high catalytic activity (62% of naphthalene **2a**). The X-ray photoemission spectroscopy (XPS) analysis shows that the Pd^2+^/Pd^0^ ratio decreases progressively with further activation and reaction treatment. These findings suggest that Pd(II) is gradually consumed during the reaction, progressively converted to Pd(0), as we work under reductive conditions.

With these results in hand, the proposed mechanism is shown in Scheme 4c. Initially, hydrazine is activated to produce H_2_ on the surface of the Pd/C heterogeneous catalyst.^48,49,56^ Intermediate α-tetralone II couples with the hydrazine in excess to form the correspondent hydrazone species.^55^ The regenerated Pd rearomatizes intermediate III to naphthylamine IV.^55,57,58^ In this step, ammonia is formed too, and it can react either with TFA in solution to give the correspondent salt or with α-tetralone II to give more naphthylamine IV.^59,60^ The plausible last step is the C–N bond cleavage promoted by TFA.^50^

## Conclusions

In summary, the presented work is the first example of HDO of phenols and naphthols catalyzed by Pd/C assessing hydrazone as the intermediate. This method not only provides a versatile pathway for HDO but also highlights the potential for more practical chemical processes without the direct employing of molecular hydrogen. Mechanistic insights revealed that hydrazine plays a dual role: selectively hydrogenating the phenolic compound and forming hydrazone after. Once this is formed, it is converted to the corresponding arylamine, thanks to a rearomatization process together with a N–N cleavage promoted by the Pd action. Finally, a C–N cleavage gives the desired HDO product. The catalytic activity of Pd/C was characterized by different analyses, and to prove the feasibility of the new methods, a consistent substrate scope of various naphthols and phenols, including pharmaceutically relevant molecules (e.g., paracetamol and β-estradiol), is presented.

## Experimental Section

### General Remarks

All reactions were carried out under an atmosphere of argon unless otherwise stated. All reported reaction temperatures corresponded to oil bath temperatures. Solvents and reagents were purchased from Sigma-Aldrich and Ambeed Chemical Company and were used without further purification unless otherwise specified. Pd/C (5 wt %) was purchased from Sigma-Aldrich (Cat. no.: 205680) and heated at 140 °C under vacuum for 1 h before running the reaction. 1-Naphthol (Cat. no.: 33420), 1-hydroxy-2-naphthoic acid (Cat. no.: 109630), 4-hydroxycarbazole (Cat. no.: 543896), 4-aminophenol (Cat. no.: A71328), 2-aminophenol (Cat. no.: A71301), 4-ethylphenol (Cat. no.: E44205), 2-ethylphenol (Cat. no.: E44000), 4-phenylphenol (Cat. no.: 134341), paracetamol (Cat. no.: P0300000), and β-estradiol (Cat. no.: E2758) were purchased from Sigma-Aldrich. 2-Methyl-1-naphthol (Cat. no.: A102087), 4-methyl-1-naphthol (Cat. no.: A645734), 4-fluoro-1-naphthol (Cat. no.: A169771), and 6-methoxy-1-naphthol (Cat. no.: A147613) were purchased from Ambeed. Dealkaline lignin (lignosulfonate) was purchased from TCI (Cat. Number: L0045). 1,4-Dioxane was purified with the Pure Solvent MD-7 purification system (Innovative Technology). 4 Å molecular sieves were purchased from Sigma-Aldrich Chemical Co. and were freshly activated in the oven for 12 h at 380 °C prior to use. Product purifications were performed either with column chromatography on a Biotage Isolera One automated chromatography system on silica gel or with preparative analytical thin-layer chromatography (TLC) using E. Merck silica gel 60 F254 precoated plates (0.25 mm). Nuclear magnetic resonance (^1^H and ^13^C) spectra were recorded on a Bruker AV500 equipped with a 60-position Sample Xpress sample changer (^1^H, 500 MHz; ^13^C, 125 MHz). Chemical shifts are expressed in parts per million (ppm) units downfield from TMS, with the solvent residue peak as the chemical shift standard (CDCl_3_: δ 7.26 ppm in ^1^H NMR, δ 77.16 ppm in ^13^C NMR; DMSO-*d*_6_ δ 2.50 ppm in ^1^H NMR, δ 39.52 ppm in ^13^C NMR; acetone-d6 δ 2.04 ppm in ^1^H NMR, δ 206.3 ppm in ^13^C NMR). Data are reported as follows: chemical shift, multiplicity (s = singlet, d = doublet, dd = doublet of doublets, t = triplet, td = triplet of doublets, q = quartet, quint = quintet, sext = sextet, sept = septet, m = multiplet, br = broad singlet), coupling constants J (Hz), and integration. All NMR spectra were recorded at room temperature. Initial catalytic tests were analyzed with a GC/FID 5975C Agilent series equipped with a capillary column DB-5MS (30 m, 0.32 mm), an FID detector, and helium as a gas carrier. EI-MS spectra were obtained using an Agilent GC–MS system. High-resolution mass spectrometry was conducted by using atmospheric pressure chemical ionization (APCI) or electrospraying ionization (ESI) performed by McGill University on a Thermo-Scientific Exactive Orbitrap. Protonated/deprotonated molecular ions or sodium adducts were used for empirical formula confirmation. The bright-field transmission electron microscopy (TEM) images were obtained on a FEI Tecnai G2 F20 scanning/transmission electron microscope at an accelerating voltage of 200 kV. The high-angle annular dark-field scanning transmission electron microscopy (HAADF-STEM) characterization was carried out on a Hitachi HD2700 Cs-corrected STEM instrument, which was used with a cold field emitter operated at 200 kV and with an electron beam diameter of ∼0.1 nm. The SEM was carried out on an FEI Quanta 450 Environment scanning electron microscopy (FE-ESEM) instrument. All reactions are stirred magnetically unless otherwise specified. Short-packed column chromatography was performed with SiliCycle SiliaFlash silica gel F60 (230–400 mesh) or Biotage Sfär silica HC D 20 μm. Flash column chromatography was performed with an Isolera TM Prime advanced automatic flash purification system.

*Caution*: hydrazine monohydrate and hydrazine in THF (1M) are potentially hazardous, and pressure can be built up at a high temperature. Therefore, appropriate personal protection should be performed while running this transformation.

## Experimental Procedures

### General Procedure for the Hydrodeoxygenation of Phenols with Hydrazine

In an oven-dried 10 mL Schlenk pressure tube, equipped with a magnetic stir bar, Pd/C (15 wt %, 15 mol %, 0.03 mmol, 61.4 mg) is added. Then, the tube is sealed with a rubber septum, linked to a high-vacuum pump, stirred, and heated at 140 °C for 1 h to preactivate the catalyst. Afterward, phenolic compound (0.2 mmol) is added under argon, and three cycles of evacuation/backfill with argon are performed. Subsequently, dioxane (0.25 mL), N_2_H_4_ in THF [1.0 M] (2.0 equiv., 0.4 mmol, 0.4 mL) or N_2_H_4_·H_2_O (3.0 equiv., 0.6 mmol, 29 μL), and TFA (1 equiv., 0.2 mmol, 15.4 μL) are added to the mixture under Ar. Ar is essential since the reaction is air sensitive. The vessel is then heated at 170 °C for 24 h under stirring. At the end of the reaction, the mixture is passed through a short column of silica gel with EtOAc to remove the catalyst.

The test using molecular hydrogen (H_2_) has been carried out preactivating Pd/C (15 wt %, 15 mol %, 0.03 mmol, 61.4 mg) at 140 °C for 1h, adding 1-naphthol (0.2 mmol, 28.8 mg) followed by three cycles of evacuation/backfill with Ar. After, dioxane (0.25 mL) and TFA (1 equiv., 0.2 mmol, 15.4 μL) are added to the mixture. Finally, a balloon filled with H_2_ is attached to the vessel. After 24 h at 170 °C under stirring, the reaction mixture is passed through a short column of silica gel with EtOAc to remove the catalyst.

### General Procedure for the Hydrodeoxygenation of Lignin

In an oven-dried 10 mL Schlenk pressure tube equipped with a magnetic stir bar, Pd/C (15 wt %, 0.03 mmol, 61.4 mg) is added. Then, the tube is sealed with a rubber septum, linked to a high-vacuum pump, stirred, and heated at 140 °C for 1 h to preactivate the catalyst. Afterward, lignin (50 mg) is added under argon, and three cycles of evacuation/backfill with argon are performed. Subsequently, dioxane (0.25 mL), N_2_H_4_ in THF [1.0 M] (0.4 mmol, 0.4 mL), and TFA (0.2 mmol, 15.4 μL) are added to the mixture under Ar. Ar is essential since the reaction is air sensitive. The vessel is then heated at 170 °C for 24 h under stirring. At the end of the reaction, the mixture is passed through a short column of silica gel with EtOAc to remove the catalyst.

### Catalyst Recycling

In an oven-dried 10 mL Schlenk pressure tube, equipped with a magnetic stir bar, Pd/C (15 wt %, 15 mol %, 0.03 mmol, 61.4 mg) is added. Then, the tube is sealed with a rubber septum, linked to a high-vacuum pump, stirred, and heated at 140 °C for 1 h to preactivate the catalyst. Afterward, 1-naphthol (0.2 mmol, 28.8 mg) is added under Ar, and three cycles of evacuation/backfill with Ar are performed. Subsequently, dioxane (0.25 mL), N_2_H_4_ in THF [1.0 M] (2.0 equiv., 0.4 mmol, 0.4 mL) or N_2_H_4_·H_2_O (3.0 equiv., 0.6 mmol, 29 μL), and TFA (1 equiv., 0.2 mmol, 15.4 μL) are added to the mixture under Ar. The vessel is then heated at 170 °C for 24 h under stirring. At the end of the reaction, Pd/C is filtered off of the mixture using a Hirsch funnel and washed with EtOAc (2 mL). The recovered catalyst is dried at 130 °C under vacuum for 6 h and reused without significant change in weight. The reaction is run under the same conditions as described before obtaining 62% of naphthalene **2a** where the rest is either the tetraline **3a** (15%) and not-reacted 1-naphthol **1a** (12%).

## Supporting Information Available

General remarks; experimental procedures (general procedure for the hydrodeoxygenation of phenols, general procedure for the hydrodeoxygenation of lignin, and catalyst recycling); catalysts screening in basic conditions; catalysts screening in acidic conditions; scope of the reaction; kinetic studies; numerical analyses of kinetic data; TEM and SEM analyses; additional mechanistic studies; and spectral data and ^1^H NMR and ^13^C NMR spectra of isolated compounds (PDF)

## Abbreviations

HDO: hydrodeoxygenation
CTH: catalytic transfer hydrogenation/hydrogenolysis
LOHCs: liquid organic hydrogen carriers
TCT: 2,4,6-trichloro-1,3,5-triazine
HOME: hydrazone as organometallic equivalent
TFA: trifluoroacetic acid
SEM: scanning electron microscopy
TEM: transition electron microscopy
XPS: X-ray photoemission spectroscopy
