# Supplementary material for: Accessing Arenes via the Hydrodeoxygenation of Phenolic Derivatives Enabled by Hydrazine
Source: ACS Catal. 2025 Feb 10;15(4):3367–76. doi: 10.1021/acscatal.4c06061 (PMC11851789; doi:10.1021/acscatal.4c06061)

SUPPORTING INFORMATION FILE

Accessing Arenes via Hydrodeoxygenation of Phenolic Derivatives Enabled by Hydrazine

Benedetta Di Erasmo,[a,b] Inna Perepichka,[b] Hui Su,[b] Chao-Jun Li,*[b] Luigi Vaccaro*[a]

[a] *Laboratory of Green S.O.C. – Dipartimento di Chimica, Biologia e Biotecnologie, Università degli Studi di Perugia, Via Elce di Sotto 8, 06123 – Perugia – Italy.*

[b] *Department of Chemistry, and FRQNT Centre for Green Chemistry and Catalysis – McGill University, 801 Sherbrooke Street West, Montreal, Quebec H3A 0B8, Canada*

* Corresponding authors:
e-mail: luigi.vaccaro@unipg.it
e-mail: cj.li@mgill.ca

total number of pages: 41

total number of tables: 3, pag: S6, S7, S9

total number of schemes: 2, pag: S8, S15

total number of figures: 5, pag: S10, S11, S12, S13, S14

Table of contents:

1. General Remarks ……………………………………………………………………….…S2
2. Experimental Procedures………………………………………………………................S4
    2.1. General procedure for the hydrodeoxygenation of phenols …………….……….....S4
    2.2. General procedure for the hydrodeoxygenation of lignin …..………………………S4
    2.3. Catalyst recycling…………………..……….......……………………………..…S5
3. Catalysts screening in basic conditions………………......……......….....……................S6
4. Catalysts screening in acidic conditions……………......……..………..…..............S7
5. Scope of the reaction……………......……………………........…..………..….............S8
6. Kinetic studies……………......………………………........…..………..……..…..............S9
7. Numeric analyses of kinetic data……………......……………..…..……..…............S10
8. TEM and SEM analyses………..……...........…..........…...........….........…...................S11
9. Additional mechanistic studies……...........…....…...........................…......……….......S15
10. Spectral data of isolated compounds……...........…..........................…......……….......S16
11. ^{1}H-NMR and ^{13}C-NMR spectra of isolated compounds……...........…........……….......S29

1. General Remarks

All reactions were carried out under an atmosphere of argon, unless otherwise stated. All reported reaction temperatures corresponded to oil bath temperatures. Solvents and reagents were purchased from Sigma-Aldrich and Ambeed chemical companies and were used without further purification unless otherwise specified. Pd/C (5 wt%) was purchased from Sigma-Aldrich (Cat. No.: 205680) and heated at 140°C under vacuum for 1 h before running the reaction. 1-naphthol (Cat. No.: 33420), 1-hydroxy-2-naphthoic acid (Cat. No.: 109630), 4-hydroxycarbazole (Cat. No.: 543896), 4-aminophenol (Cat. No.: A71328), 2-aminophenol (Cat. No.: A71301), 4-ethylphenol (Cat. No.: E44205), 2-ethylphenol (Cat. No.: E44000), 4-phenylphenol (Cat. No.: 134341), paracetamol (Cat.

No.: P0300000) β-estradiol (Cat. No.: E2758) were purchased from Sigma-Aldrich. 2-methyl-1-naphthol (Cat. No.: A102087), 4-methyl-1-naphthol (Cat. No.: A645734), 4-fluoro-1-naphthol (Cat. No.: A169771), 6-methoxy-1-naphthol (Cat. No.: A147613) were purchase from Ambeed. Dealkaline lignin (lignosulfonate) was purchased from TCI (Cat. Number: L0045). 1,4-Dioxane was purified by the Pure Solvent MD-7 purification system (Innovative Technology). 4 Å molecular sieves were purchased from Sigma-Aldrich chemical company and were freshly activated in the oven for 12 h at 380°C prior to use. Product purifications were performed either with column chromatography on a Biotage Isolera One automated chromatography system on silica gel or with preparative analytical thin-layer chromatography (TLC) using E. Merck silica gel 60 F254 pre-coated plates (0.25 mm). Nuclear magnetic resonance (^{1}H and ^{13}C) spectra were recorded on a Bruker AV500 equipped with a 60-position Sample Xpress sample changer (^{1}H, 500 MHz; ^{13}C, 125 MHz). Chemical shifts are expressed in parts per million (ppm) units downfield from TMS, with the solvent residue peak as the chemical shift standard ($CDCl_3$: δ 7.26 ppm in ^{1}H NMR, δ 77.16 ppm in ^{13}C NMR; DMSO-d6 δ 2.50 ppm in ^{1}H NMR, δ 39.52 ppm in ^{13}C NMR; acetone-d6 δ 2.04 ppm in ^{1}H NMR, δ 206.3 ppm in ^{13}C NMR). Data are reported as following: chemical shift, multiplicity (s = singlet, d = doublet, dd = doublet of doublets, t = triplet, td = triplet of doublets, q = quartet, quint = quintet, sext = sextet, sept = septet, m = multiplet, br = broad singlet), coupling constants J (Hz), and integration. All NMR spectra were recorded at room temperature. Initial catalytic tests were analysed with a GC/FID 5975C Agilent series equipped with a capillary column DB-5MS (30 m, 0.32 mm), an FID detector, and helium as a gas carrier. EI-MS was obtained from the Agilent GC-MS system. High-resolution mass spectrometry was conducted by using atmospheric pressure chemical ionization (APCI) or electro-spraying ionization (ESI) performed by McGill University on a Thermo-Scientific Exactive Orbitrap. Protonated/deprotonated molecular ions or sodium adducts were used for empirical formula confirmation. The bright field transmission electron microscopy (TEM) images were obtained on FEI Tecnai G2 F20 S/TEM at accelerating voltage of 200 kV. The high-angle annular dark-field scanning transmission electron microscopy (HAADF-STEM) characterization was carried out on a Hitachi

HD2700 Cs-corrected STEM, which was used with a cold field emitter operated at 200 kV and with an electron beam diameter of ~ 0.1 nm. The scanning electron microscopy (SEM) was carried out on a FEI Quanta 450 Environment scanning electron microscopy (FE-ESEM). All reactions are stirred magnetically unless otherwise specified. Short-packed column chromatography was performed with Silicycle SiliaFlash silica gel F60 (230–400 mesh) or Biotage Sfär silica HC D 20 μm. Flash column chromatography was performed with IsoleraTM Prime advanced automatic flash purification system.

Caution: hydrazine monohydrate and hydrazine in THF (1M) are potentially hazardous, and pressure can be built up at high temperature. Therefore, appropriate personal protections should be performed running this transformation.

2. Experimental procedures

2.1. General procedure for the hydrodeoxygenation of phenols

In an oven-dried 10 mL Schlenk pressure tube, equipped with a magnetic stir bar Pd/C (15 wt%, 15 mol%, 0.03 mmol, 61.4 mg) is added. Then, the tube is sealed with a rubber septum, linked to a high-vacuum pump, stirred and heated at 140 °C for 1 h to pre-activate the catalyst. Afterwards, phenolic compound (0.2 mmol) is added under Argon (Ar) and three cycles of evacuation/backfill with Ar are performed. Subsequently, dioxane (0.25 mL), N_2H_4 in THF [1.0 M] (2.0 equiv., 0.4 mmol, 0.4 mL) or $N_2H_4 \cdot H_2O$ (3.0 equiv., 0.6 mmol, 29 μL) and TFA (1 equiv., 0.2 mmol, 15.4 μL) are added to the mixture under Ar. Ar is essential since the reaction is air sensitive. The vessel is then heated at 170 °C for 24 h under stirring. At the end of the reaction the mixture is passed through a short column of silica gel with EtOAc to remove the catalyst.

The test using molecular hydrogen (H_2) has been carried out always pre-activating Pd/C (15 wt%, 15 mol%, 0.03 mmol, 61.4 mg) at 140°C for 1h, adding 1-naphthol (0.2 mmol, 28.8 mg) followed by three cycles of evacuation/backfill with Ar. After, dioxane (0.25 mL) and TFA (1 equiv., 0.2 mmol, 15.4 μL) are added to the mixture. Finally, a balloon filled with H_2 has been attached to the

vessel.After 24 h at 170°C under stirring, the reaction mixture is passed through a short column of silica gel with EtOAc to remove the catalyst.

2.2. General procedure for the hydrodeoxygenation of lignin

In an oven-dried 10 mL Schlenk pressure tube, equipped with a magnetic stir bar Pd/C (15 wt%, 0.03 mmol, 61.4 mg) is added. Then, the tube is sealed with a rubber septum, linked to a high-vacuum pump, stirred and heated at 140 °C for 1 h to pre-activate the catalyst. Afterwards, dealkaline lignin (50 mg) is added under argon and three cycles of evacuation/backfill with argon are performed. Subsequently, dioxane (0.25 mL), N_2H_4 in THF [1.0 M] (0.4 mmol, 0.4 mL) and TFA (0.2 mmol, 15.4 μL) are added to the mixture under Ar. Ar is essential since the reaction is air sensitive. The vessel is then heated at 170 °C for 24 h under stirring. At the end of the reaction the mixture is passed through a short column of silica gel with EtOAc to remove the catalyst.

2.3. Catalyst recycling

In an oven-dried 10 mL Schlenk pressure tube, equipped with a magnetic stir bar Pd/C (15 wt%, 15 mol%, 0.03 mmol, 61.4 mg) is added. Then, the tube is sealed with a rubber septum, linked to a high-vacuum pump, stirred and heated at 140 °C for 1 h to pre-activate the catalyst. Afterwards, 1-naphthol (0.2 mmol, 28.8 mg) is added under Ar and three cycles of evacuation/backfill with Ar are performed. Subsequently, dioxane (0.25 mL), N_2H_4 in THF [1.0 M] (2.0 equiv., 0.4 mmol, 0.4 mL) or $N_2H_4{\cdot}H_2O$ (3.0 equiv., 0.6 mmol, 29 μL) and TFA (1 equiv., 0.2 mmol, 15.4 μL) are added to the mixture under Ar. The vessel is then heated at 170 °C for 24 h under stirring. At the end of the reaction, Pd/C was filtered off from the mixture using a Hirsh funnel and washed with EtOAc (2 mL). The recovered catalyst was dried at 130°C under vacuum for 6 h and reused without significant change in weight. The reaction was run under the same conditions as described before obtaining 62% of naphthalene **2a** where the rest is either the tetraline **3a** (15%) and not-reacted 1-naphthol **1a** (12%).

3. Catalysts screening in basic conditions

Table S1. Catalyst screening in basic conditions. [a]

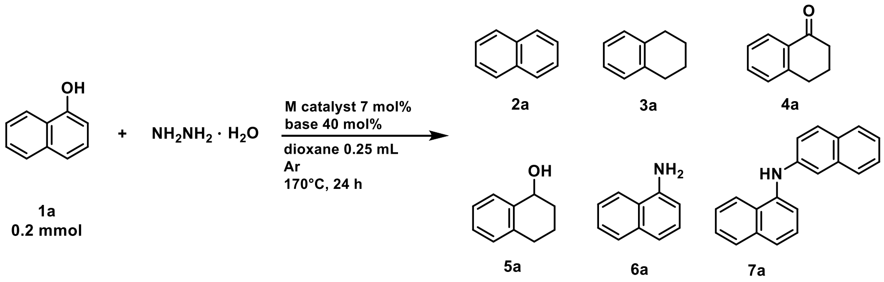

Entry	$N_2H_4 \cdot H_2O$ amount (equiv)	Catalyst	Additive	1a GC conv (%) [b]	Selectivity (2a:3a:4a:5a:6a:7a) [b]
1	4.5	Pd/C 7 mol%	*t*-BuOLi 40 mol%	90	3:3:1:2:90:0
2	6.0	Pd/C 7 mol%	*t*-BuOLi 40 mol%	95	5:0:0:0:92:3
3	4.5	Rh/C 7 mol%	*t*-BuOLi 40 mol%	91	9:17:8:17:5:44:0
4	4.5	$[Rh(I)(ept)Cl]_2$ 7 mol%	LiOH 40 mol%	65	7:3:38:23:15:14
5	4.5	Pd/Al_2O_3 7 mol%	*t*-BuOLi 40 mol%	64	5:2:0:0:95:0
6	6.0	Pd/Al_2O_3 7 mol%	*t*-BuOLi 40 mol%	53	5:2:0:0:95:0
7	6.0	$[Rh(I)(oct)Cl]_2$ 7 mol%	*t*-BuOLi 40 mol%	21	14:0:14:37:34:0
8	6.0	$Rh(I)(oct)_2BF_4$ 7 mol%	*t*-BuOLi 40 mol%	0	-
9	6.0	$[Rh(I)(oct)(OH)]_2$ 7mol%	*t*-BuOLi 40 mol%	17	0:0:0:100:0:0
10	6.0	$Ru(oct)Cl_2$ 7 mol%	*t*-BuOLi 40 mol%	0	-
11	4.5	Pd/C 7 mol% + Rh/C 7 mol%	*t*-BuOLi 40 mol%	79	5:13:17:13:42:0
12	6.0	Pd/C 7 mol% + Rh/C 7 mol%	*t*-BuOLi 40 mol%	42	13:0:0:5:82:0
13	6.0	Pd/C 5 mol% + Rh/C 3 mol%	t-BuOLi 40 mol%	80	19:10:0:4:67:0
14	6.0	Pd/C 3 mol% + Ru-PNN 3 mol%	*t*-BuOLi 40 mol%	0	-

[a] Reaction conditions: **1a** (0.2 mmol), **2** , catalyst, base 40 mol%, dioxane 0.25 mL, 24 h, 170°C, Ar, 10 mL pressure tube. [b] Results obtained by calibration curve method.

4. Catalysts screening in acidic conditions

Table S2. Catalyst screening in acidic conditions. [a]

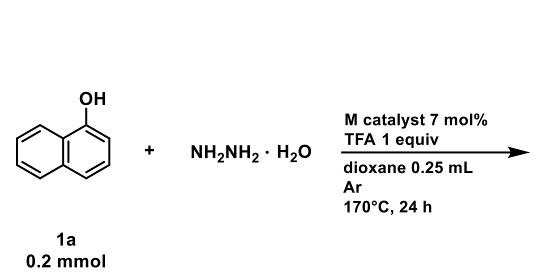

Entry	$N_2H_4 \cdot H_2O$ amount (equiv)	Catalyst	Additive	1a GC conv (%) [b]	Selectivity (2a:3a:4a:5a:7a) [b]
1	3.0	Pd/C 7 mol%	TFA 1 equiv	78	68:11:0:2:13
2 [c]	3.0	Pd/C 7 mol%	TFA 1 equiv	77	65:12:7:7:9
3 [d]	3.0	Pd/C 7 mol%	TFA 1 equiv	20	58:0:12:0:30
4	3.0	Pd/C 3 mol% + Rh/C 5 mol%	TFA 1 equiv	99	51:37:0:7:6
5	3.0	Rh/C 7 mol%	TFA 1 equiv	82	10:34:0:22:33
6	3.0	Pd/C 3 mol% + Ru-PNN 3 mol%	TFA 1 equiv	0	-
7	3.0	Pd/CaCO3 7 mol%	TFA 1 equiv	0	-
8	3.0	PdO_2 7 mol%	TFA 1 equiv	0	-
9	3.0	$Pd(OH)_2/C$ 15 mol%	TFA 1 equiv	95	49:32:4:6:8
10	3.0	$Ni(cod)_2$ 7 mol%	TFA 1 equiv	6	100:0:0:0:0
11	3.0	Pd/C 7 mol% + $Ni(cod)_2$ 7 mol%	TFA 1 equiv	35	40:0:0:0:60

[a] Reaction conditions: **1a** (0.2 mmol), **2 ,** catalyst, TFA 1 equiv, dioxane 0.25 mL, 24 h, 170°C, Ar, 10 mL pressure tube [b] Results obtained by calibration curve method. [c] dioxane 0.4 mL [d] dioxane 1.0 mL

5. Scope of the reaction

Other commercial naphthols and phenols were used in the developed protocol without obtaining the arenes of interest. Here we have reported nonreactive substrates in this process. It is evident that oxygen-containing heterocycles, as well as phenols with electron-withdrawing groups and significant steric hindrance, are adversely affected by these reaction conditions.

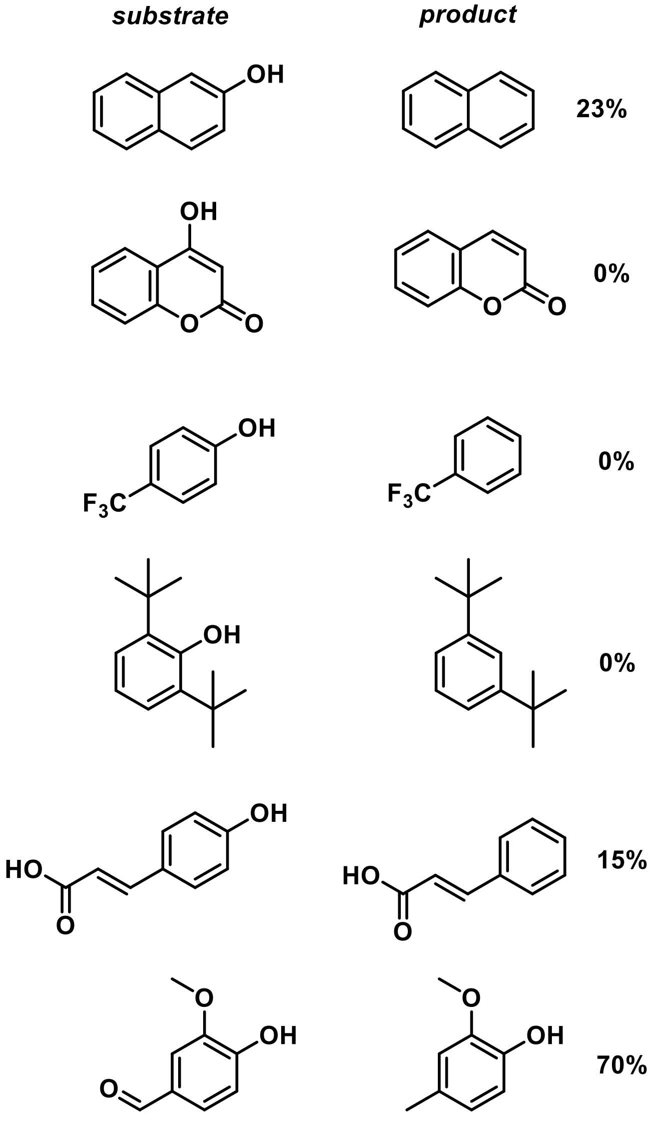

Scheme S1. Unsuccessful substrates for the HDO *via* hydrazone chemistry. NMR yields are provided.

6. Kinetic studies

Table S3. Kinetic studies.

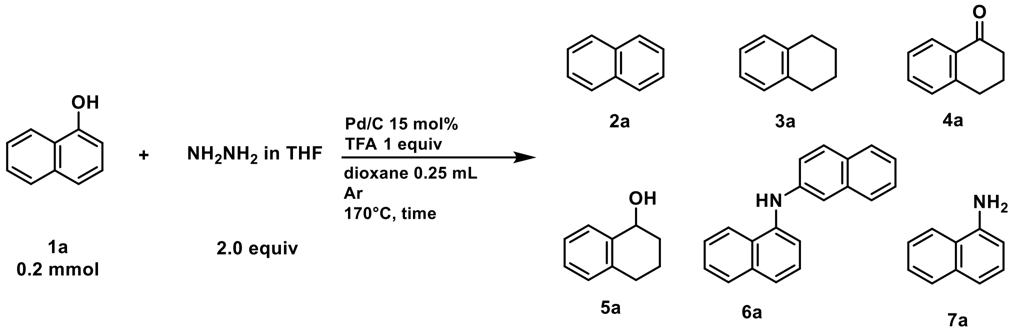

Entry	Time	1a	2a	3a	4a	5a	6a	7a
1	5 min	93	0	0	0	0	0	0
2	15 min	70	0	0	7	3	0	20
3	30 min	58	0	2	12	0	0	13
4	1 h	51	6	0	3	6	3	23
5	2,5 h	45	20	7	3	6	3	7
6	7 h	31	30	5	2	4	6	8
7	10 h	13	36	11	0	2	5	6
8	14 h	15	59	11	0	0	7	6
9	19 h	10	63	13	0	0	8	6
10	24 h	11	70	9	0	0	7	3
11	41 h	0	58	6	0	0	10	5

[a] Reaction conditions: **1a** (0.2 mmol), **2 ,** Pd/C 15 mol%, N_2H_4 in THF [1.0 M] 2 equiv., TFA 1 equiv, dioxane 0.25 mL, 24 h, 170°C, Ar, 10 mL pressure tube. NMR yields are reported using dibromomethane as I.S

7. Numeric analyses of kinetic data

1-naphthol curve can be divided in 2 kinetics:

1) until 5 hours the 1-naphthol curve follows a 1st kinetic order trend

$$[1a] = [1a]_0 e^{-kt}$$ → k = 1/t1 = 1200,188 → k = $8,3*10^{-4}$ M^{-1}

2) after 1-naphthol curve reaches a plateau because, being in heterogeneous conditions, all the possible active sites can be saturated.

Figure S1. Kinetic data for 1-naphthol.

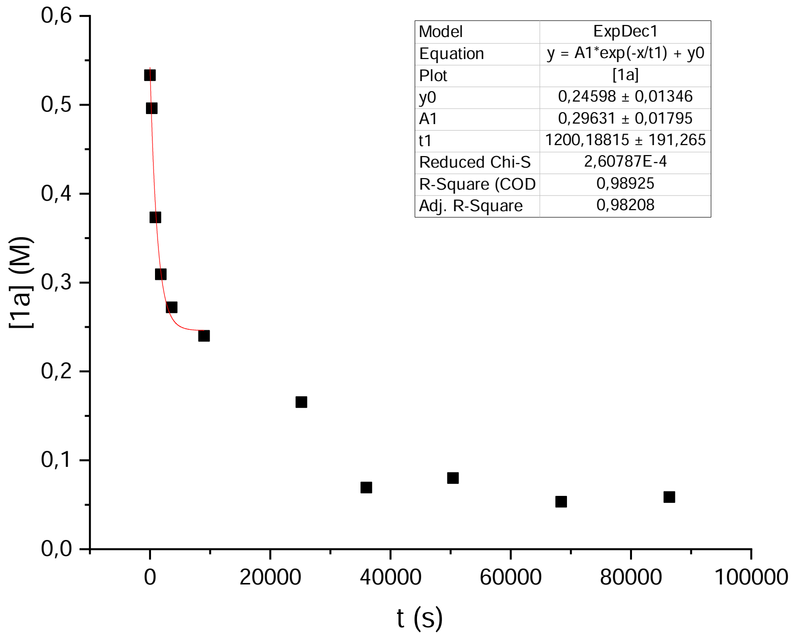

8. TEM and SEM analyses

Figure S2. SEM (a) and TEM (b) analyses of the fresh catalyst Pd/C prior pre-activation.

a) SEM analysis

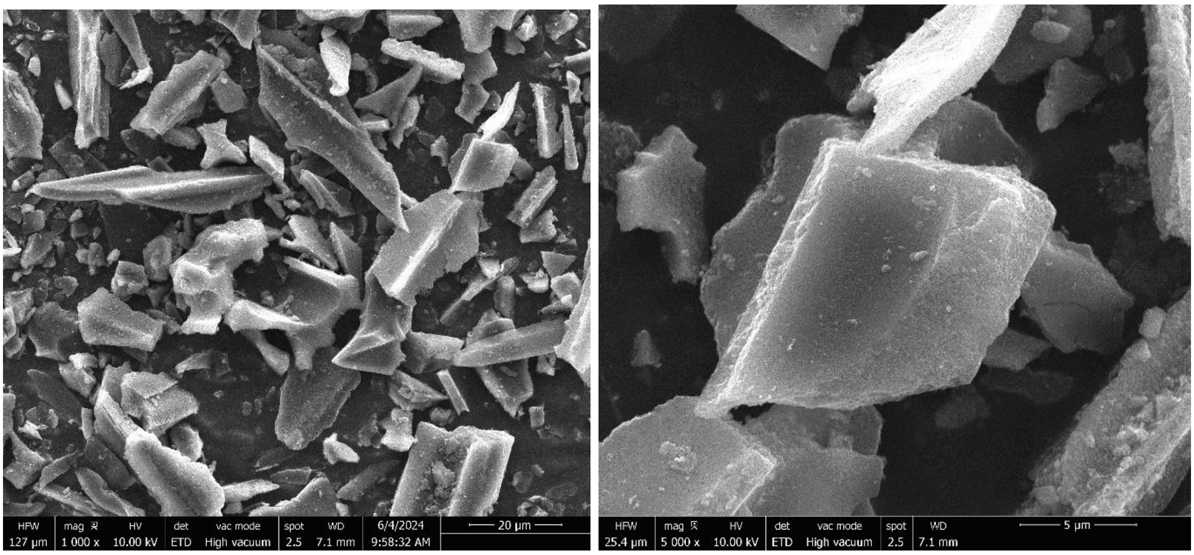

b) TEM analysis

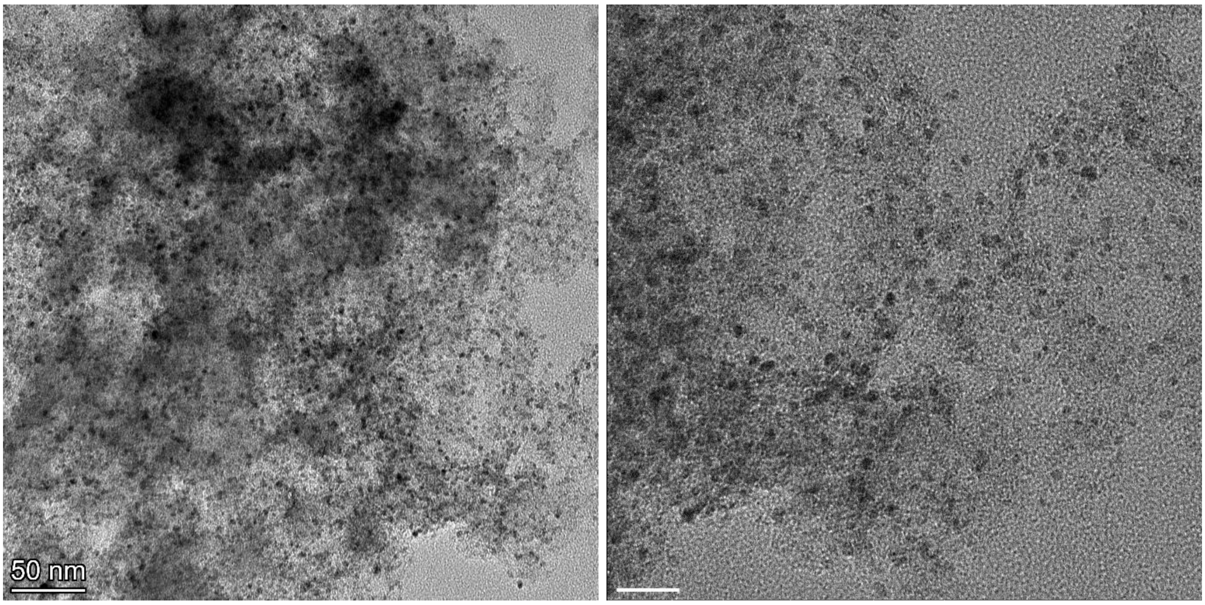

Figure S3. SEM (a) and TEM (b) analyses of the activated Pd/C. No change in morfology and size is observed after the pre-activation.

a) SEM analysis

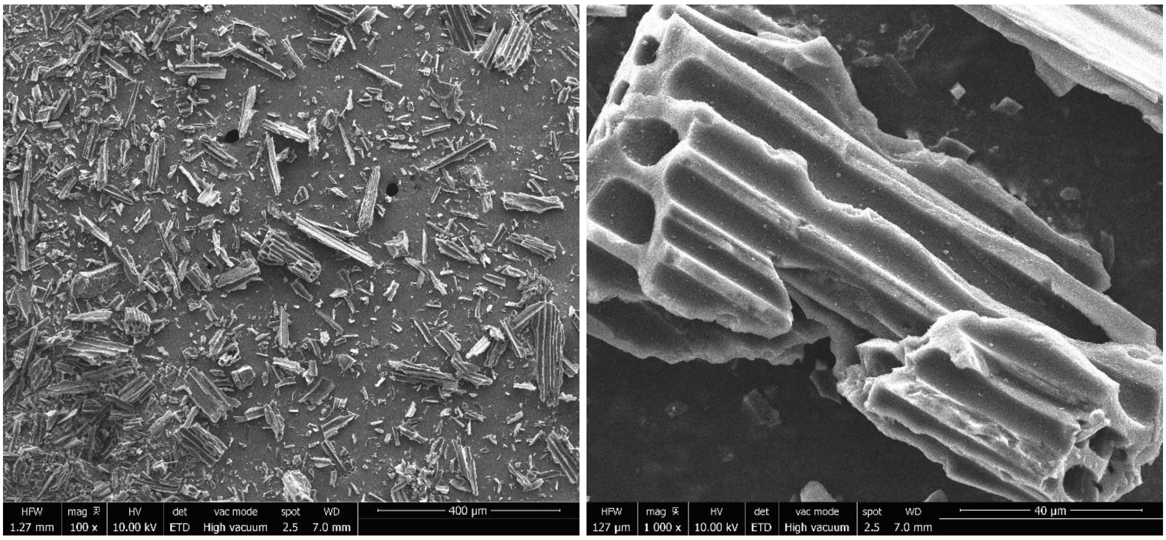

b) TEM analysis

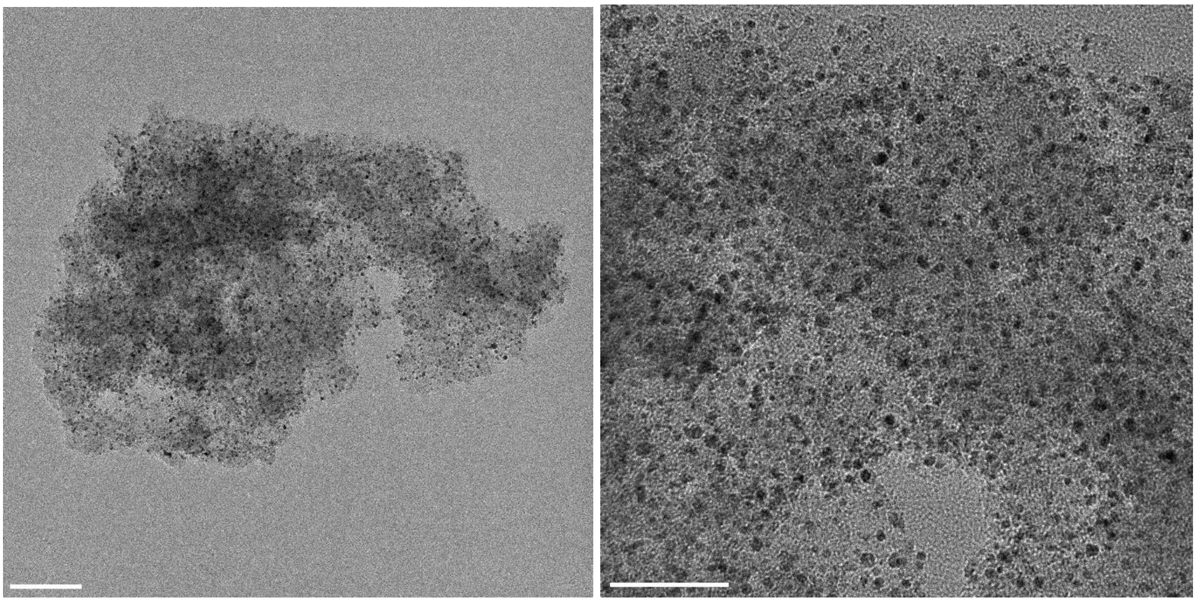

Figure S4. SEM (a) and TEM (b) analyses of Pd/C after 3h reaction time. During the reaction, no particular change in morfology and size is observed.

a) SEM analysis

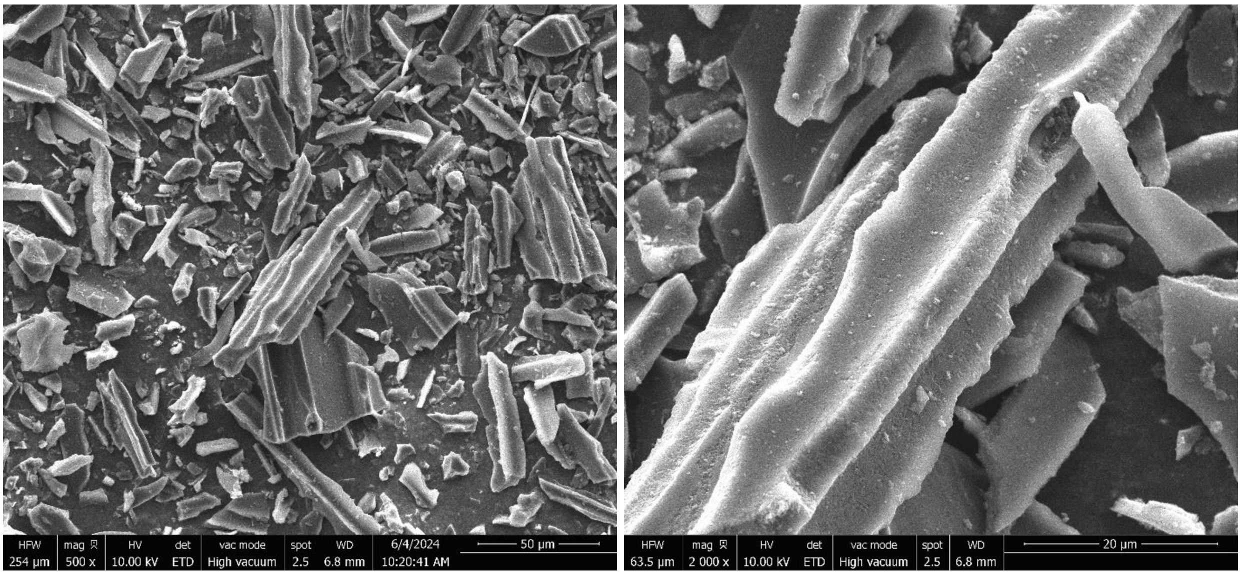

b) TEM analysis

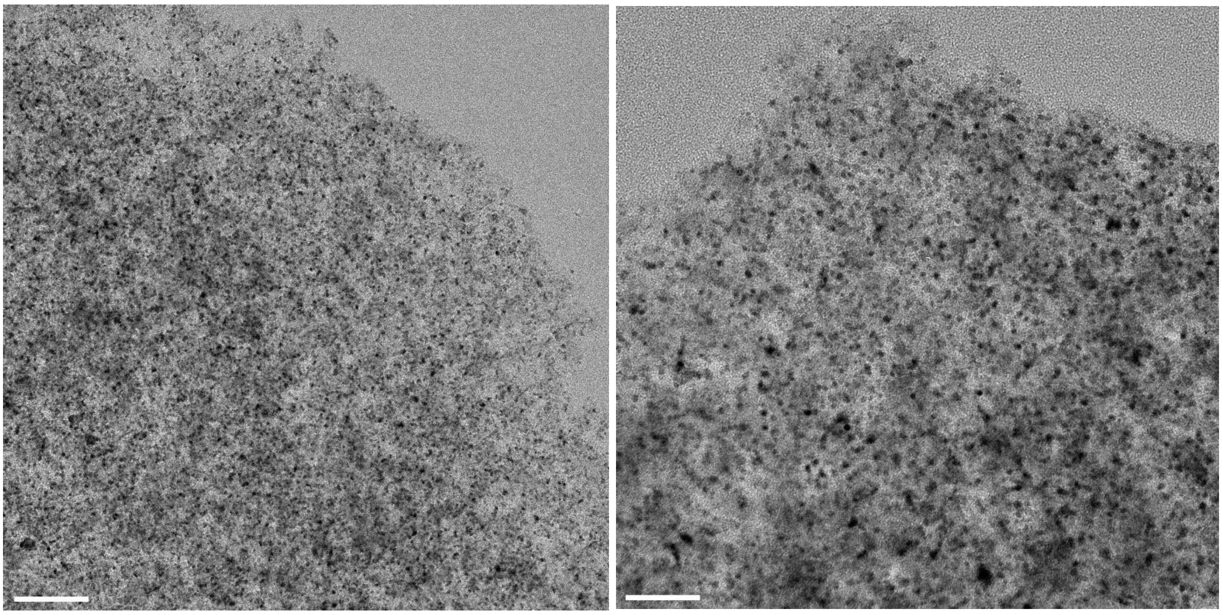

Figure S5. SEM (a) and TEM (b) analyses of Pd/C after 24h reaction time. After the reaction, no change in morfology and size is observed. The catalyst mantains its properties.

a) SEM analysis

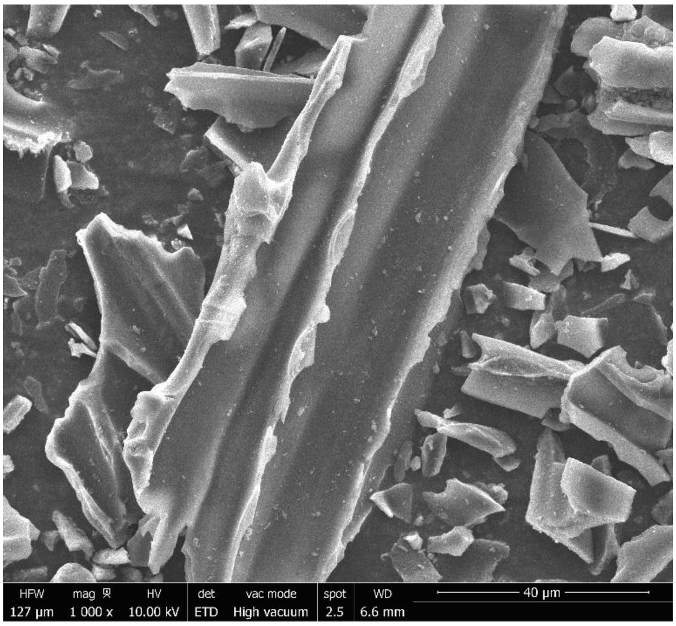

b) TEM analysis

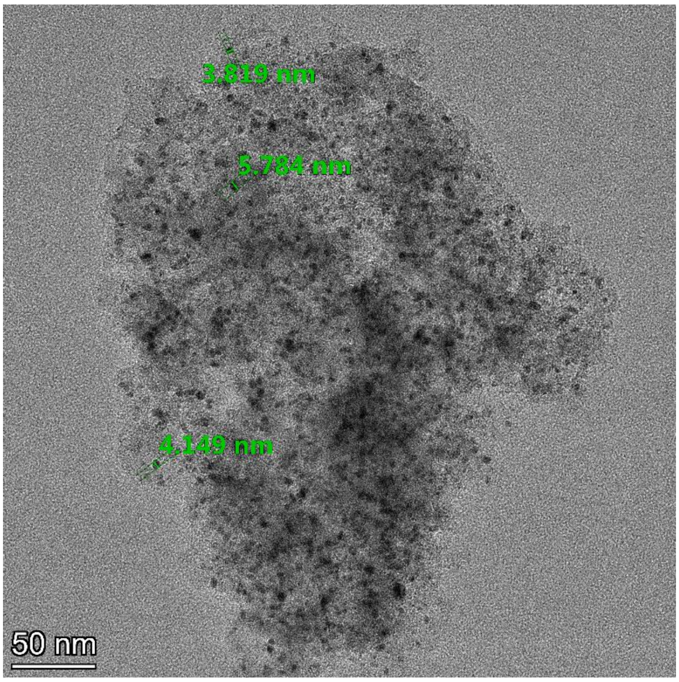

9. Additional mechanistic studies

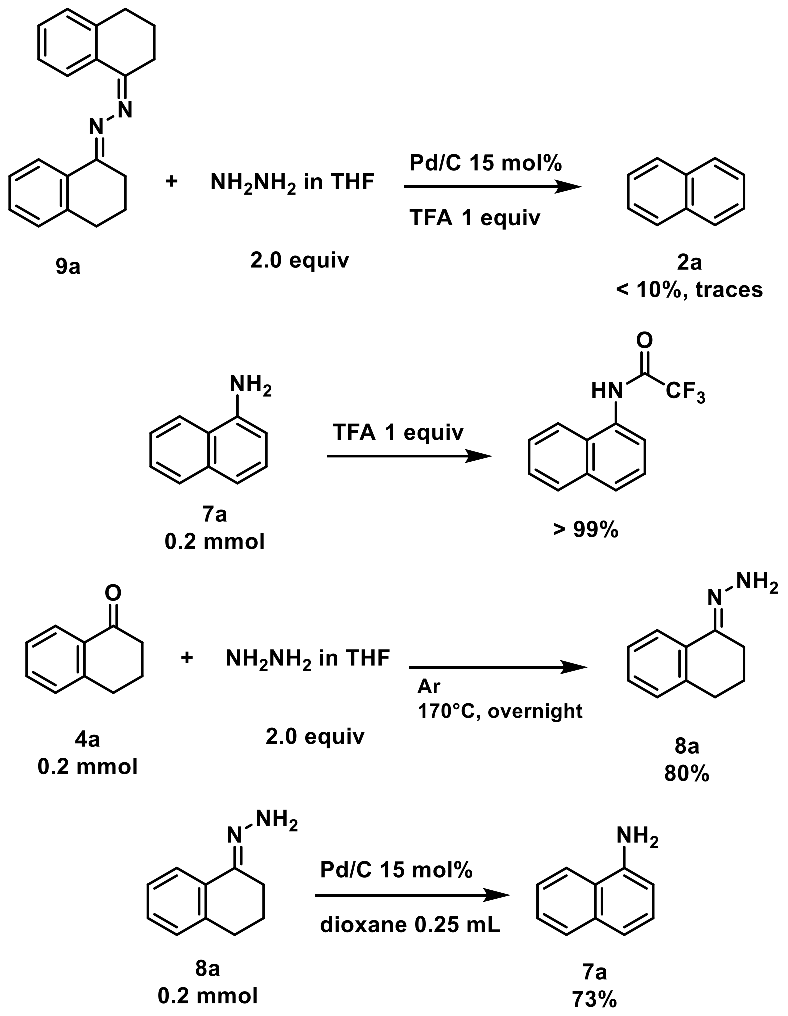

Scheme S2. Additional mechanistic studies.

10. Spectral data of isolated compounds

**Chem. Name**	**naphthalene (2a)**
**Lit. Ref.**	S. Kato, Y. Saga, M. Kojima, H. Fuse, S. Matsunaga, A. Fukatsu, M. Kondo, S. Masaoka, M. Kana, *J. Am. Chem. Soc.* **2017**, *139*, 2204−2207, https://doi.org/10.1021/jacs.7b00253

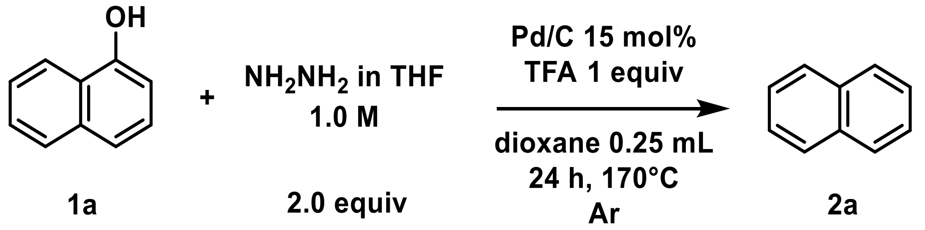

METHOD:

In an oven-dried 10 mL Schlenk pressure tube, equipped with a magnetic stirbar Pd/C (15 wt%, 15 mol%, 0.03 mmol, 61.4 mg) is added. Then, the tube is sealed with a rubber septum and linked to a high-vacuum pump and it is heated at 140 °C for 1 h to activate the catalyst. After 1-naphthol (0.2 mmol, 28.8 mg) is added under Argon and three cycles of evacuation/backfill with Argon are performed. Subsequently, dioxane (0.25 mL), N_2H_4 in THF [1.0 M] (2.0 equiv., 0.4 mmol, 0.4 mL) and TFA (1 equiv., 0.2 mmol, 15.4 µL) are added to the mixture under Ar. The vessel is then heated at 170 °C for 24 h under stirring. At the end of the reaction the mixture is passed through a pad of silica gel to remove the heterogeneous catalyst with EtOAc. The filtrate is then purified with a TLC preparative with pentane as the eluent. The purified product is obtained as a white solid. (isolated yield 70%, 17 mg).

**Mol Formula**		$C_{10}H_8$	**m.p.**	79°C–80°C
	**δ value**	**No. H**	**Mult.**	**J value/Hz**
**^{1}H NMR 500 MHz $CDCl_3$**	7.86-7.84	4	*m*	
	7.50-7.48	4	*m*	

^{13}C NMR (126 MHz, $CDCl_3$) δ : 133.5, 127.9, 125.8

HRMS (APCI): calculated for 128.0621 (M); found: 128.0625

Chem. Name	**2-methyl-naphthalene (2b)**
**Lit. Ref.**	S. Kato, Y. Saga, M. Kojima, H. Fuse, S. Matsunaga, A. Fukatsu, M. Kondo, S. Masaoka, M. Kana, *J. Am. Chem. Soc.* **2017**, *139*, 2204−2207, https://doi.org/10.1021/jacs.7b00253

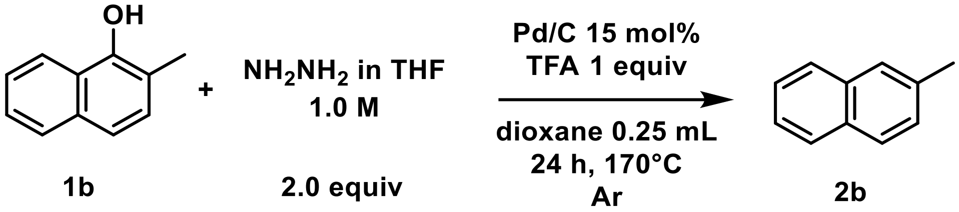

METHOD:

In an oven-dried 10 mL Schlenk pressure tube, equipped with a magnetic stirbar Pd/C (15 wt%, 15 mol%, 0.03 mmol, 61.4 mg) is added. Then, the tube is sealed with a rubber septum and linked to a high-vacuum pump, and it is heated at 140 °C for 1 h to activate the catalyst. After 2-methyl-1-naphthol (0.2 mmol, 31.6 mg) is added under Argon and three cycles of evacuation/backfill with Argon are performed. Subsequently, dioxane (0.25 mL), N_2H_4 in THF [1.0 M] (2.0 equiv., 0.4 mmol, 0.4 mL) and TFA (1 equiv., 0.2 mmol, 15.4 µL) are added to the mixture under Ar. The vessel is then heated at 170 °C for 24 h under stirring. At the end of the reaction the mixture is passed through a pad of silica gel to remove the heterogeneous catalyst with EtOAc. The filtrate is then purified with a TLC preparative with hexane as the eluent. The purified product is obtained as a pale yellow liquid. (isolated yield 52%, 15 mg).

**Mol Formula**	$C_{11}H_{10}$	**m.p.**	Pale yellow liquid

	**δ value**	**No. H**	**Mult.**	**J value/Hz**
**^{1}H NMR 500 MHz $CDCl_3$**	7.81-7.74	3	*m*	-
	7.62	1	*s*	-
	7.46-7.39	2	*m*	-
	7.32	1	*d*	8.2
	2.52	3	*s*	-

^{13}C NMR (126 MHz, $CDCl_3$) δ : 135.4, 133.7, 131.7, 128.1, 127.7, 127.6, 127.2, 126.8, 125.9, 124.9, 21.7

HRMS (APCI+): calculated for 141.0699 (M+); found: 141.0706

Chem. Name	**1-methyl-naphthalene (2c)**
**Lit. Ref.**	S. Kato, Y. Saga, M. Kojima, H. Fuse, S. Matsunaga, A. Fukatsu, M. Kondo, S. Masaoka, M. Kana, *J. Am. Chem. Soc.* **2017**, *139*, 2204−2207, https://doi.org/10.1021/jacs.7b00253

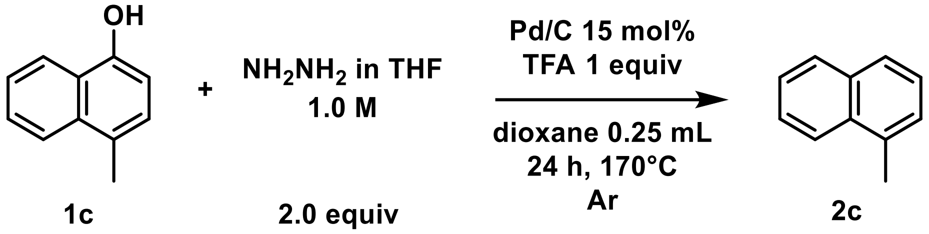

METHOD:

In an oven-dried 10 mL Schlenk pressure tube, equipped with a magnetic stirbar Pd/C (15 wt%, 15 mol%, 0.03 mmol, 61.4 mg) is added. Then, the tube is sealed with a rubber septum and linked to a high-vacuum pump and it is heated at 140 °C for 1 h to activate the catalyst. After 4-methyl-1-naphthol (0.2 mmol, 31.6 mg) is added under Argon and three cycles of evacuation/backfill with Argon are performed. Subsequently, dioxane (0.25 mL), N_2H_4 in THF [1.0 M] (2.0 equiv., 0.4 mmol, 0.4 mL) and TFA (1 equiv., 0.2 mmol, 15.4 µL) are added to the mixture under Ar. The vessel is then heated at 170 °C for 24 h under stirring. At the end of the reaction the mixture is passed through a pad of silica gel to remove the heterogeneous catalyst with EtOAc. The filtrate is then purified with a TLC preparative with hexane as the eluent. The purified product is obtained as a yellow liquid. (isolated yield 53%, 15 mg).

**Mol Formula**		$C_{11}H_{10}$	**m.p.**	Yellow liquid
	**δ value**	**No. H**	**Mult.**	**J value/Hz**
**^{1}H NMR 500 MHz $CDCl_3$**	8.02	1	*d*	8.4
	7.87	1	*m*	-
	7.73	1	*d*	8.1
	7.56-7.49	2	*m*	
	7.41-7.38	1	*m*	-
	7.35-7.34	1	*m*	-
	2.72	3	*s*	

^{13}C NMR (126 MHz, $CDCl_3$) δ :134.3, 133.6, 132.6, 128.5, 126.6, 126.4, 125.7, 125.6, 125.5, 124.1, 19.4

HRMS (APCI+): calculated for 141.0699 (M+); found: 141.0694

Chem. Name	2-methoxy-naphthalene (2d)
Lit. Ref.	H. Yue, L. Guo, S.-C. Lee, X. Liu, M. Rueping, *Angew. Chem. Int. Ed.* **2017**, *56*, 3972–3976, https://doi.org/10.1002/ange.201612624

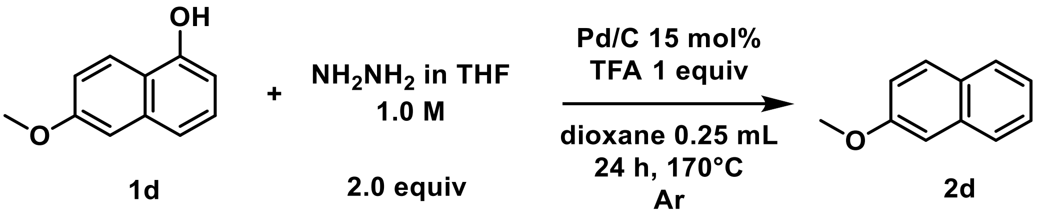

METHOD:

In an oven-dried 10 mL Schlenk pressure tube, equipped with a magnetic stirbar Pd/C (15 wt%, 15 mol%, 0.03 mmol, 61.4 mg) is added. Then, the tube is sealed with a rubber septum and linked to an high-vacuum pump and it is heated at 140 °C for 1 h to activate the catalyst. After, 6-methoxy-1-naphthol (0.2 mmol, 34.8 mg) is added under Argon and three cycles of evacuation/backfill with Argon are performed. Subsequently, dioxane (0.25 mL), N_2H_4 in THF [1.0 M] (2.0 equiv., 0.4 mmol, 0.4 mL) and TFA (1 equiv., 0.2 mmol, 15.4 µL) are added to the mixture under Ar. The vessel is then heated at 170 °C for 24 h under stirring. At the end of the reaction the mixture is passed through a pad of silica gel to remove the heterogeneous catalyst with EtOAc. The filtrate is then purified with a TLC preparative with hexane:ethyl acetate 9:1 as the eluent. The purified products is obtained as a white solid. (isolated yield 41%, 13 mg).

**Mol Formula**		$C_{11}H_{10}O$	**m.p.**	75°C-76°C
**^{1}H NMR 500 MHz $CDCl_3$**	**δ value**	**No. H**	**Mult.**	**J value/Hz**
	7.78-7.76	1	*m*	-
	7.75-7.73	2	*m*	-
	7.46-7.42	1	*m*	-
	7.35-7.32	1	*m*	-
	7.16-7.14	2	*m*	-
	3.93	3	*s*	-

^{13}C NMR (126 MHz, $CDCl_3$) δ : 157.6, 134.7, 129.4, 129.0, 127.6, 126.7, 126.4, 123.6, 118.7, 105.8, 55.3

HRMS (APCI+): calculated for 159.0804 (M+H); found: 159.0811

Chem. Name	**naphthalene (2a)**
**Lit. Ref.**	S. Kato, Y. Saga, M. Kojima, H. Fuse, S. Matsunaga, A. Fukatsu, M. Kondo, S. Masaoka, M. Kana, *J. Am. Chem. Soc.* **2017**, *139*, 2204−2207, https://doi.org/10.1021/jacs.7b00253

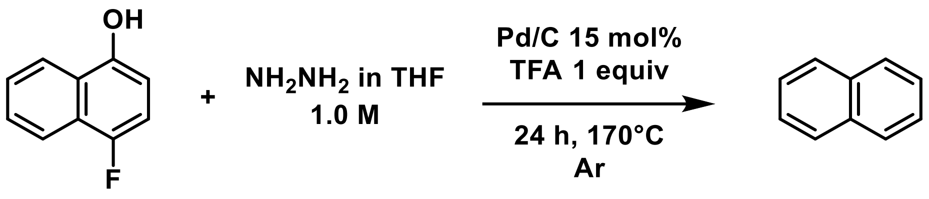

1e **2.0 equiv** **2a**

METHOD:

In an oven-dried 10 mL Schlenk pressure tube, equipped with a magnetic stirbar Pd/C (15 wt%, 15 mol%, 0.03 mmol, 61.4 mg) is added. Then, the tube is sealed with a rubber septum and linked to an high-vacuum pump and it is heated at 140 °C for 1 h to activate the catalyst. After 4-fluoro-1-naphthol (0.2 mmol, 32.4 mg) is added under Argon and three cycles of evacuation/backfill with Argon are performed. Subsequently, dioxane (0.25 mL), N_2H_4 in THF [1.0 M] (2.0 equiv., 0.4 mmol, 0.4 mL) and TFA (1 equiv., 0.2 mmol, 15.4 μL) are added to the mixture under Ar. The vessel is then heated at 170 °C for 24 h under stirring. At the end of the reaction the mixture is passed through a pad of silica gel to remove the heterogeneous catalyst with EtOAc. The filtrate is then purified with a TLC preparative with pentane as the eluent. The purified product is obtained as a white solid. (isolated yield 17%, 4 mg).

**Mol Formula**		$C_{10}H_8$	**m.p.**	79°C–80°C
**^{1}H NMR 500 MHz $CDCl_3$**	**δ value**	**No. H**	**Mult.**	**J value/Hz**
	7.86-7.84	4	*m*	
	7.49-7.47	4	*m*	

^{13}C NMR (126 MHz, $CDCl_3$) δ : 133.5, 127.9, 125.8

HRMS (APCI): calculated for 128.0621 (M); found: 128.0625

Chem. Name	naphthalene (2a)
Lit. Ref.	S. Kato, Y. Saga, M. Kojima, H. Fuse, S. Matsunaga, A. Fukatsu, M. Kondo, S. Masaoka, M. Kana, *J. Am. Chem. Soc.* **2017**, *139*, 2204−2207, https://doi.org/10.1021/jacs.7b00253

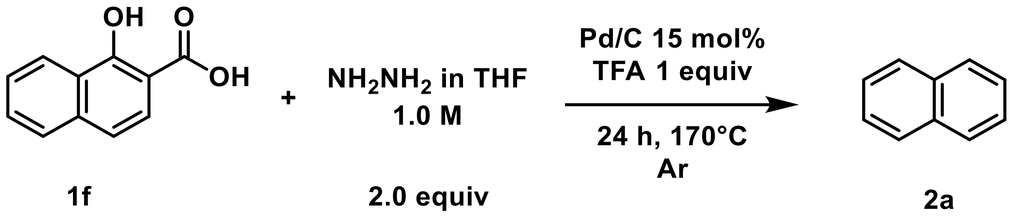

METHOD:

In an oven-dried 10 mL Schlenk pressure tube, equipped with a magnetic stirbar Pd/C (15 wt%, 15 mol%, 0.03 mmol, 61.4 mg) is added. Then, the tube is sealed with a rubber septa and linked to an high-vacuum pump and it is heated at 140 °C for 1 h to activate the catalyst. After 1-hydroxy-2-naphthoic acid (0.2 mmol, 37.6 mg) is added under Argon and three cycles of evacuation/backfill with Argon are performed. Subsequently, dioxane (0.25 mL), N_2H_4 in THF [1.0 M] (2.0 equiv., 0.4 mmol, 0.4 mL) and TFA (1 equiv., 0.2 mmol, 15.4 µL) are added to the mixture under Ar. The vessel is then heated at 170 °C for 24 h under stirring. At the end of the reaction the mixture is passed through a pad of silica gel to remove the heterogeneous catalyst with EtOAc. The filtrate is then purified with a TLC preparative with pentane as the eluent. The purified product is obtained as a white solid. (isolated yield 26%, 7 mg).

**Mol Formula**	$C_{10}H_8$	**m.p.**	79°C–80°C

	**δ value**	**No. H**	**Mult.**	**J value/Hz**
**^{1}H NMR 500 MHz $CDCl_3$**	7.86-7.84	4	*m*	
	7.50-7.48	4	*m*	

^{13}C NMR (126 MHz, $CDCl_3$) δ: 133.5, 127.9, 125.8

HRMS (APCI): calculated for 128.0621 (M); found: 128.0625

Chem. Name	carbazole (2g)
Lit. Ref.	Alimi, R. Remy, C. G. Bochet, *Eur. J. Org. Chem.* **2017**, 3197–3210, https://doi.org/10.1002/ejoc.201700300

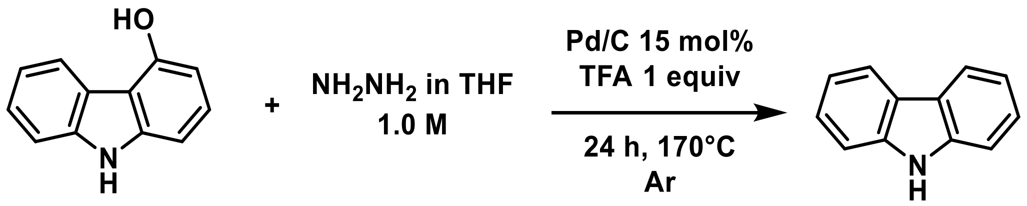

1g　　2.0 equiv　　2g

METHOD:

In an oven-dried 10 mL Schlenk pressure tube, equipped with a magnetic stirbar Pd/C (15 wt%, 15 mol%, 0.03 mmol, 61.4 mg) is added. Then, the tube is sealed with a rubber septa and linked to an high-vacuum pump and it is heated at 140 °C for 1 h to activate the catalyst. After 4-hydroxycarbazole (0.2 mmol, 36.7 mg) is added under Argon and three cycles of evacuation/backfill with Argon are performed. Subsequently, N_2H_4 in THF [1.0 M] (2.0 equiv., 0.4 mmol, 0.4 mL) and TFA (1 equiv., 0.2 mmol, 15.4 µL) are added to the mixture under Ar. The vessel is then heated at 170 °C for 24 h under stirring. At the end of the reaction the mixture is passed through a pad of silica gel to remove the heterogeneous catalyst with EtOAc. The filtrate is then purified with a TLC preparative with hexane as the eluent. The purified product is obtained as a yellow solid. (isolated yield 40%, 13 mg).

**Mol Formula**	$C_{12}H_9N$	**m.p.**	240°C-246°C

	**δ value**	**No. H**	**Mult.**	**J value/Hz**
**^{1}H NMR 500 MHz CD_3COCD_3**	10.32	0.5	*s, br*	-
	8.13	2	*d*	7.8
	7.53-7.52	2	*m*	-
	7.41-7.38	2	*m*	-
	7.21-7.18	2	*m*	-

^{13}C NMR (126 MHz, CD_3COCD_3) δ: 125.5, 123.1, 120.0, 118.7, 110.81, 110.76

GC-EIMS (m/z, %): calculated for 167.0730 (M); found: 167.0734

Chem. Name	aniline
Lit. Ref.	C. Zhu, G. Li, D. H. Ess, J. R. Falck, L. Kürti, *J.Am.Chem.Soc.* **2012**, *134*, 18253−18256, https://doi.org/10.1021/ja309637r

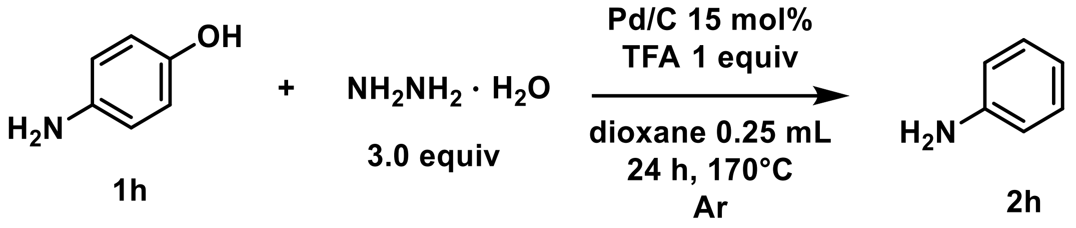

METHOD:

In an oven-dried 10 mL Schlenk pressure tube, equipped with a magnetic stirbar Pd/C (15 wt%, 15 mol%, 0.03 mmol, 61.4 mg) is added. Then, the tube is sealed with a rubber septa and linked to an high-vacuum pump and it is heated at 140 °C for 1 h to activate the catalyst. After 4-aminophenol (0.2 mmol, 21.8 mg) is added under Argon and three cycles of evacuation/backfill with Argon are performed. Subsequently, dioxane (0.25 mL), $N_2H_4 \cdot H_2O$ (3.0 equiv., 0.6 mmol, 30 µL) and TFA (1 equiv., 0.2 mmol, 15.4 µL) are added to the mixture under Ar. The vessel is then heated at 170 °C for 24 h under stirring. At the end of the reaction the mixture is passed through a pad of silica gel to remove the heterogeneous catalyst with EtOAc. The filtrate is then purified with a TLC preparative with hexane as the eluent. The purified product is obtained as a yellow liquid. (isolated yield 30%, 6 mg).

**Mol Formula**	C_6H_7N	**m.p.**	Yellow liquid

	**δ value**	**No. H**	**Mult.**	**J value/Hz**
**^{1}H NMR 500 MHz $CDCl_3$**	7.19-7.15	2	*m*	-
	6.79-6.76	1	*m*	
	6.71-6.69	2	*m*	
	3.64	2	*s, br*	

^{13}C NMR (126 MHz, $CDCl_3$) δ : 146.4, 129.3, 118.6, 115.1

GC-EIMS (m/z, %): 93 (31), 92 (9), 66 (19), 65 (9)

Chem. Name	aniline (2h)
Lit. Ref.	C. Zhu, G. Li, D. H. Ess, J. R. Falck, L. Kürti, *J.Am.Chem.Soc.* **2012**, *134*, 18253−18256, https://doi.org/10.1021/ja309637r

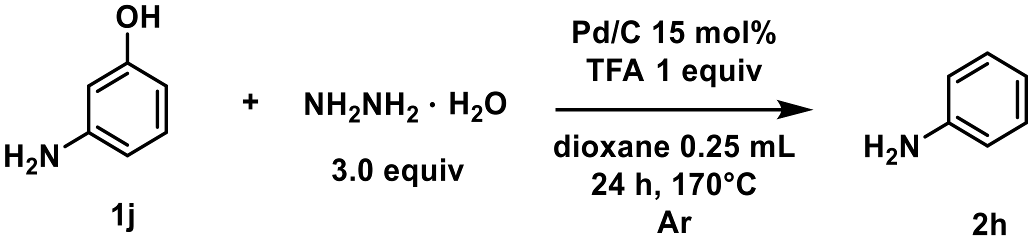

METHOD:

In an oven-dried 10 mL Schlenk pressure tube, equipped with a magnetic stirbar Pd/C (15 wt%, 15 mol%, 0.03 mmol, 61.4 mg) is added. Then, the tube is sealed with a rubber septa and linked to an high-vacuum pump and it is heated at 140 °C for 1 h to activate the catalyst. After 3-aminophenol (0.2 mmol, 21.8 mg) is added under Argon and three cycles of evacuation/backfill with Argon are performed. Subsequently, dioxane (0.25 mL), $N_2H_4{\cdot}H_2O$ (3.0 equiv., 0.6 mmol, 30 µL) and TFA (1 equiv., 0.2 mmol, 15.4 µL) are added to the mixture under Ar. The vessel is then heated at 170 °C for 24 h under stirring. At the end of the reaction the mixture is passed through a pad of silica gel to remove the heterogeneous catalyst with EtOAc. The filtrate is then purified with a TLC preparative with hexane as the eluent. The purified product is obtained as a yellow liquid. (isolated yield 17%, 2 mg).

Mol Formula		C_6H_7N	m.p.	Yellow liquid
	δ value	No. H	Mult.	J value/Hz
^{1}H NMR 500 MHz $CDCl_3$	7.18-7.14	2	*m*	-
	6.78-6.74	1	*m*	-
	6.70-6.68	2	*m*	-
	3.64	2	*s, br*	

^{13}C NMR (126 MHz, $CDCl_3$) δ : 146.4, 129.3, 118.6, 115.1

GC-EIMS (m/z, %): 93 (31), 92 (9), 66 (19), 65 (9)

Chem. Name	**ethyl-benzene (2j)**
**Lit. Ref.**	S. P. Cummings, T.-N. Le, G. E. Fernandez, L. G. Quiambao, B. J. Stokes, *J.Am.Chem.Soc.* **2016**, *138*, 6107−6110, https://doi.org/10.1021/jacs.6b02132

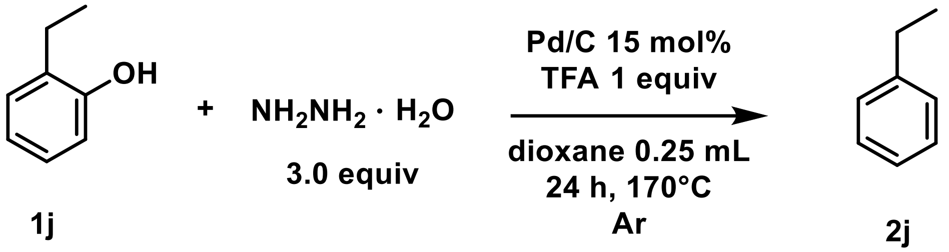

METHOD:

In an oven-dried 10 mL Schlenk pressure tube, equipped with a magnetic stirbar Pd/C (15 wt%, 15 mol%, 0.03 mmol, 61.4 mg) is added. Then, the tube is sealed with a rubber septa and linked to an high-vacuum pump and it is heated at 140 °C for 1 h to activate the catalyst. After 2-ethylphenol (0.2 mmol, 24.4 mg) is added under Argon and three cycles of evacuation/backfill with Argon are performed. Subsequently, dioxane (0.25 mL), $N_2H_4 \cdot H_2O$ (3.0 equiv., 0.6 mmol, 30 µL) and TFA (1 equiv., 0.2 mmol, 15.4 µL) are added to the mixture under Ar. The vessel is then heated at 170 °C for 24 h under stirring. At the end of the reaction the mixture is passed through a pad of silica gel to remove the heterogeneous catalyst with EtOAc. The filtrate is then purified with a TLC preparative with hexane as the eluent. The purified product is obtained as a colorless liquid. (isolated yield 17%, 2 mg).

**Mol Formula**	C_8H_{10}		**m.p.**	Colorless liquid
	**δ value**	**No. H**	**Mult.**	**J value/Hz**
	7.32-7.29	2	*m*	-
	7.23-7.18	3	*m*	-
**^{1}H NMR 500 MHz $CDCl_3$**	2.67	2	*q*	7.6 Hz
	1.27	3	*t*	7.6 Hz

^{13}C NMR (126 MHz, $CDCl_3$) δ : 144.3, 128.3, 127.9, 125.6, 28.9, 15.6

GC-EIMS (m/z, %): 106 (M+, 60), 105 (25), 91 (100), 79 (15), 78 (17), 77 (17), 67 (18), 51 (21), 39 (16), 27 (15)

Chem. Name	ethyl-benzene (2j)
Lit. Ref.	S. P. Cummings, T.-N. Le, G. E. Fernandez, L. G. Quiambao, B. J. Stokes, *J.Am.Chem.Soc.* **2016**, *138*, 6107−6110, https://doi.org/10.1021/jacs.6b02132

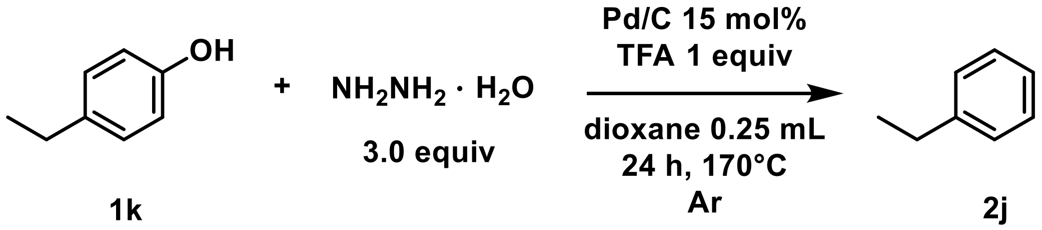

METHOD:

In an oven-dried 10 mL Schlenk pressure tube, equipped with a magnetic stirbar Pd/C (15 wt%, 15 mol%, 0.03 mmol, 61.4 mg) is added. Then, the tube is sealed with a rubber septa and linked to an high-vacuum pump and it is heated at 140 °C for 1 h to activate the catalyst. Subsequently, 4-ethylphenol (0.2 mmol, 24.4 mg), dioxane (0.25 mL), $N_2H_4{\cdot}H_2O$ (3.0 equiv., 0.6 mmol, 30 µL) and TFA (1 equiv., 0.2 mmol, 15.4 µL) are added to the mixture under Ar. The vessel is then heated at 170 °C for 24 h under stirring. At the end of the reaction the mixture is passed through a pad of silica gel to remove the heterogeneous catalyst with EtOAc. The filtrate is then purified with a TLC preparative with hexane as the eluent. The purified product is obtained as a colorless liquid. (isolated yield 37%, 6 mg).

**Mol Formula**		C_8H_{10}	**m.p.**	Colorless liquid
^{1}**H NMR 500 MHz CDCl3**	**δ value**	**No. H**	**Mult.**	**J value/Hz**
	7.32-7.29	2	*m*	-
	7.23-7.18	3	*m*	-
	2.67	2	*q*	7.6 Hz
	1.28-1.25	3	*m*	-

^{13}**C NMR (126 MHz, CDCl3) δ :** 144.3, 128.3, 127.9, 125.6, 28.9, 15.6

GC-EIMS (m/z, %): 106 (M+, 60), 105 (25), 91 (100), 79 (15), 78 (17), 77 (17), 67 (18), 51 (21), 39 (16), 27 (15)

**Chem. Name**	**1,1'-biphenyl (4g)**
**Lit. Ref.**	G. Ranjani, R. Nagarajan, *Org. Lett.* **2017**, *19*, 15, 3974–3977; https://doi.org/10.1021/acs.orglett.7b01669

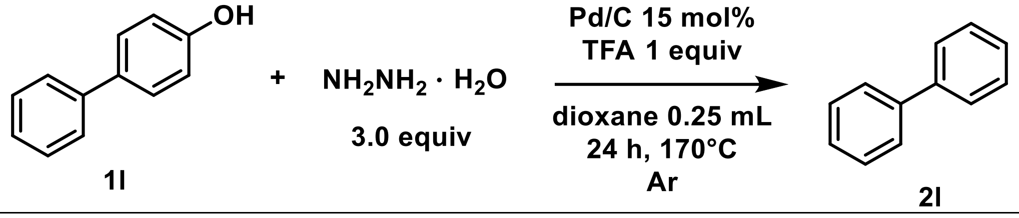

METHOD:

In an oven-dried 10 mL Schlenk pressure tube, equipped with a magnetic stirbar Pd/C (15 wt%, 15 mol%, 0.03 mmol, 61.4 mg) is added. Then, the tube is sealed with a rubber septa and linked to an high-vacuum pump and it is heated at 140 °C for 1 h to activate the catalyst. Subsequently 4-phenylphenol (0.2 mmol, 34 mg) dioxane (0.25 mL), $N_2H_4{\cdot}H_2O$ (3.0 equiv., 0.6 mmol, 30 µL) and TFA (1 equiv., 0.2 mmol, 15.4 µL) are added to the mixture under Ar. The vessel is then heated at 170 °C for 24 h under stirring. At the end of the reaction the mixture is passed through a pad of silica gel to remove the heterogeneous catalyst with EtOAc. The filtrate is then purified with a TLC preparative with hexane as the eluent. The purified product is obtained as a white solid. (isolated yield 57%, 18 mg).

**Mol Formula**		$C_{12}H_{10}$	**m.p.**	67°C–68°C
**^{1}H NMR 500 MHz $CDCl_3$**	**δ value**	**No. H**	**Mult.**	**J value/Hz**
	7.61-7.59	4	*m*	-
	7.46-7.43	4	*m*	-
	7.37-7.34	2	*m*	-

^{13}C NMR (126 MHz, $CDCl_3$) δ : 141.3, 128.8, 127.3, 127.2

GC-EIMS (m/z, %): 155 (M+1,12), 154 (M, 100), 153 (43), 152 (32), 74 (19), 63 (19), 51 (32), 50 (29)

**Chem. Name**	**aniline (2h)**
**Lit. Ref.**	C. Zhu, G. Li, D. H. Ess, J. R. Falck, L. Kürti, *J.Am.Chem.Soc.* **2012**, *134*, 18253−18256, https://doi.org/10.1021/ja309637r

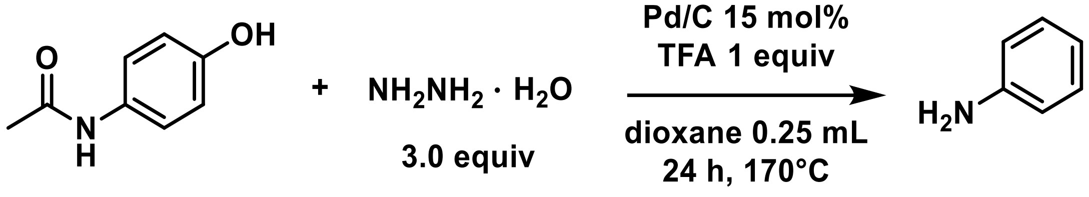

paracetamol 1m **2h**

METHOD:

In an oven-dried 10 mL Schlenk pressure tube, equipped with a magnetic stirbar Pd/C (15 wt%, 15 mol%, 0.03 mmol, 61.4 mg) is added. Then, the tube is sealed with a rubber septa and linked to an high-vacuum pump and it is heated at 140 °C for 1 h to activate the catalyst. After *N*-(4-hydroxyphenyl)acetamide (0.2 mmol, 30.2 mg) is added under Argon and three cycles of evacuation/backfill with Argon are performed. Subsequently, dioxane (0.25 mL), $N_2H_4{\cdot}H_2O$ (3.0 equiv., 0.6 mmol, 30 µL) and TFA (1 equiv., 0.2 mmol, 15.4 µL) are added to the mixture under Ar. The vessel is then heated at 170 °C for 24 h under stirring. At the end of the reaction the mixture is passed through a pad of silica gel to remove the heterogeneous catalyst with EtOAc. The filtrate is then purified with a TLC preparative with hexane as the eluent. The purified product is obtained as a yellow liquid. (isolated yield 45%, 8 mg).

**Mol Formula**	C_6H_7N	**m.p.**	Yellow liquid	
**^{1}H NMR 500 MHz $CDCl_3$**	**δ value**	**No. H**	**Mult.**	**J value/Hz**
	7.21-7.18	2	*m*	-
	6.81-6.78	1	*m*	-
	6.72-6.70	2	*m*	-
	3.65	2	*s, br*	

^{13}C NMR (126 MHz, $CDCl_3$) δ :146.5, 129.3, 118.6, 115.1

GC-EIMS (m/z, %): 94 (M+1, 18), 93 (M, 100), 92 (29), 66 (62), 65 (29)

naphthalene

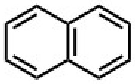

2a

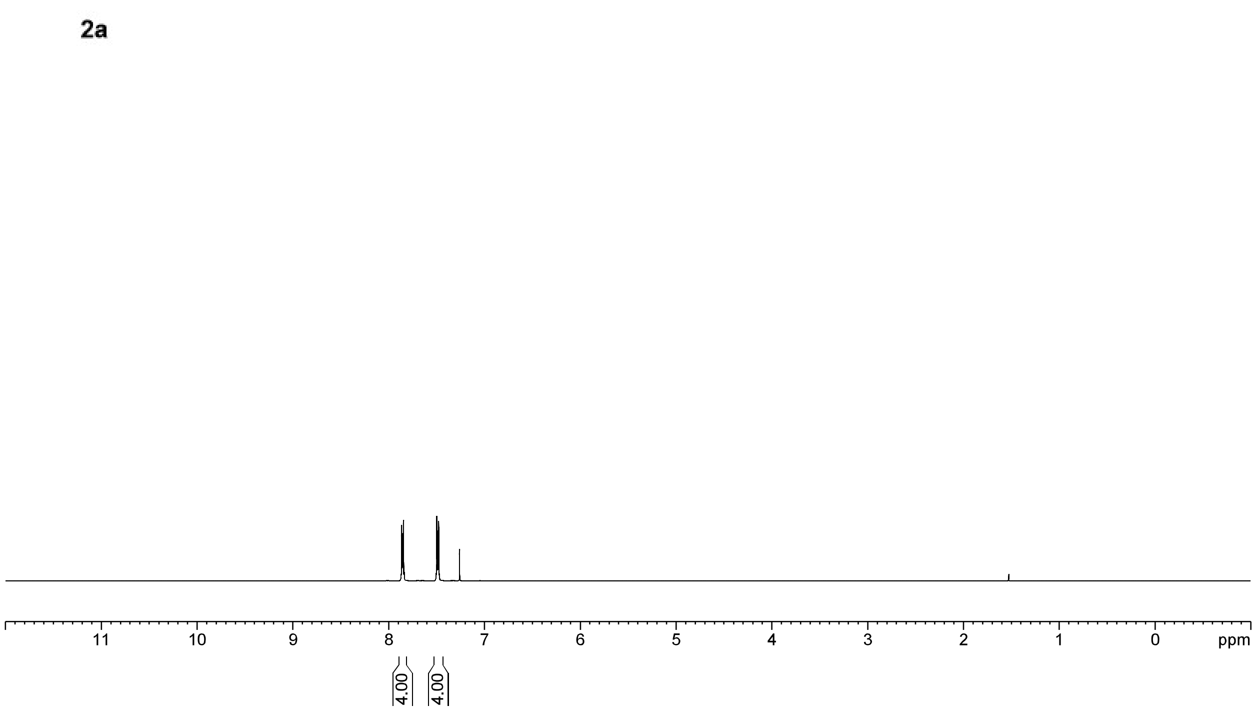

naphthalene

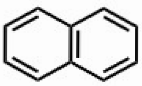

2a

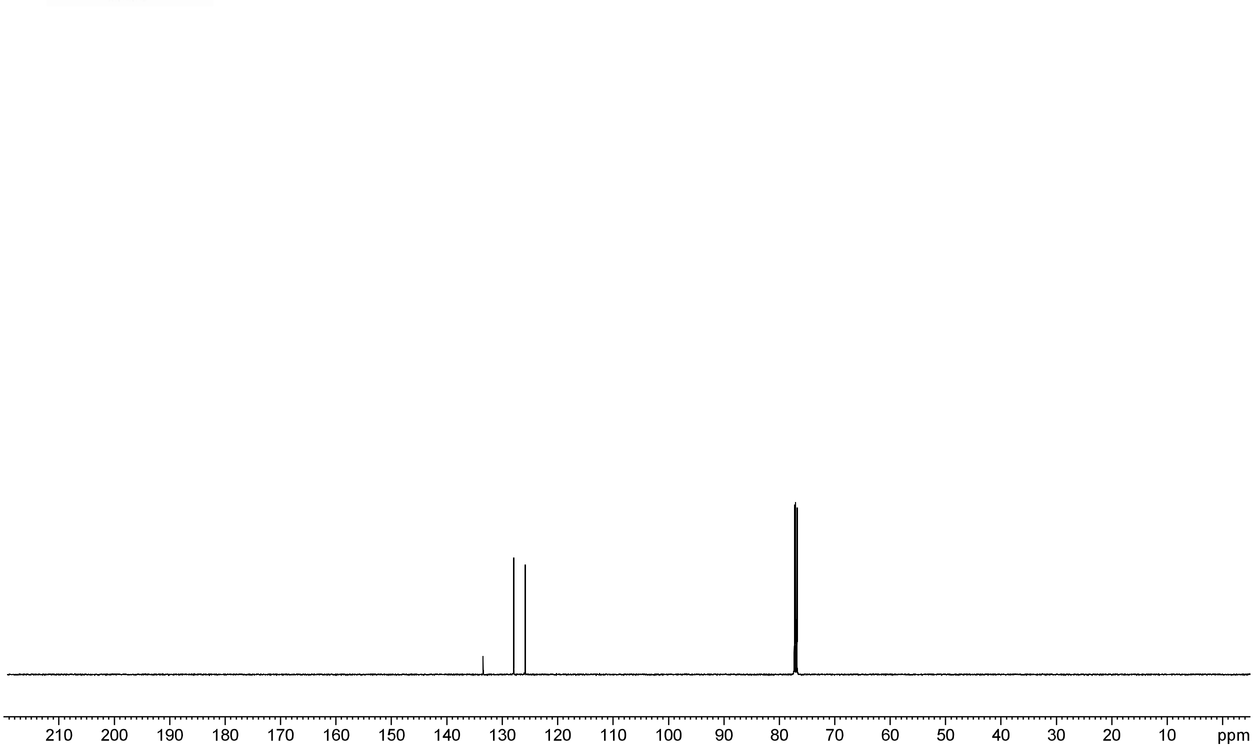

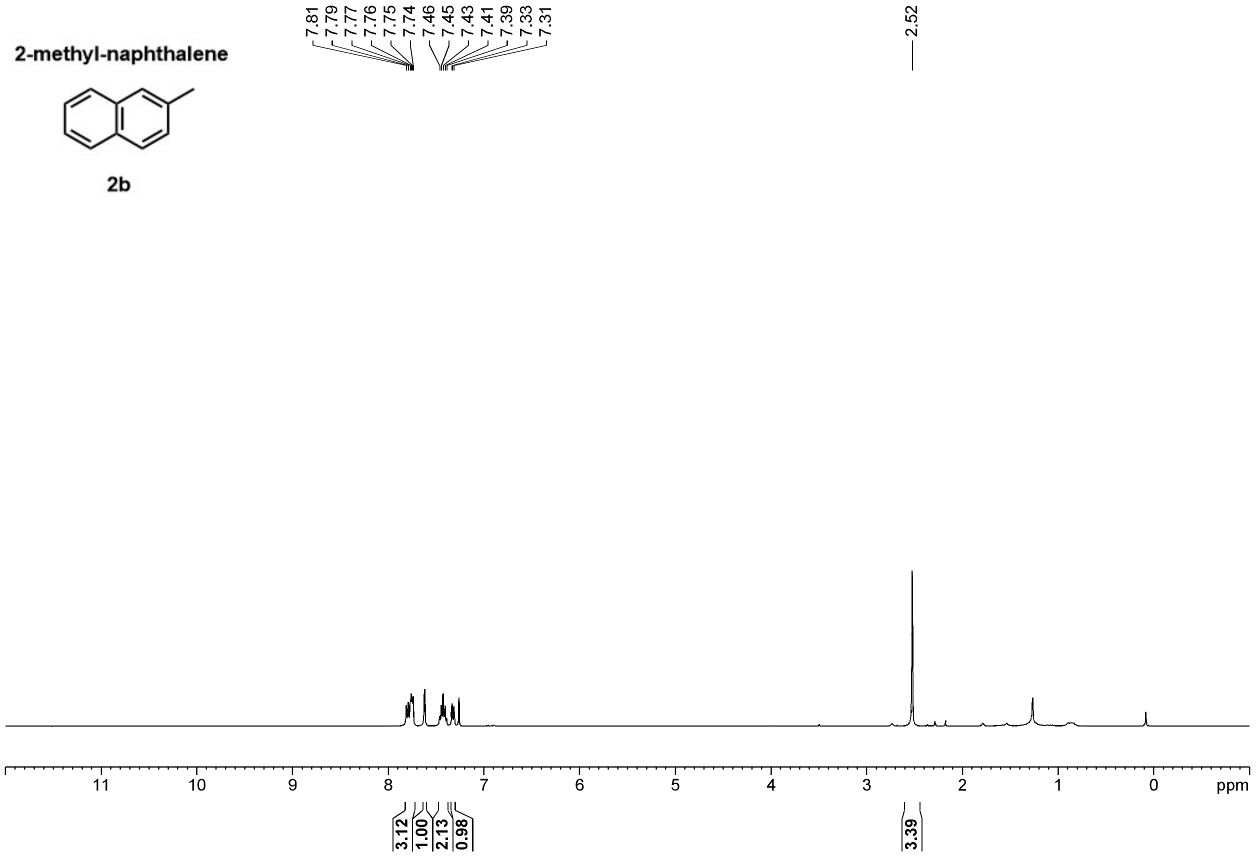
2-methyl-naphthalene
2b
7.81
7.79
7.77
7.76
7.75
7.74
7.46
7.45
7.43
7.41
7.39
7.33
7.31
2.52
11 10 9 8 7 6 5 4 3 2 1 0 ppm
3.12
1.00
2.13
0.98
3.39

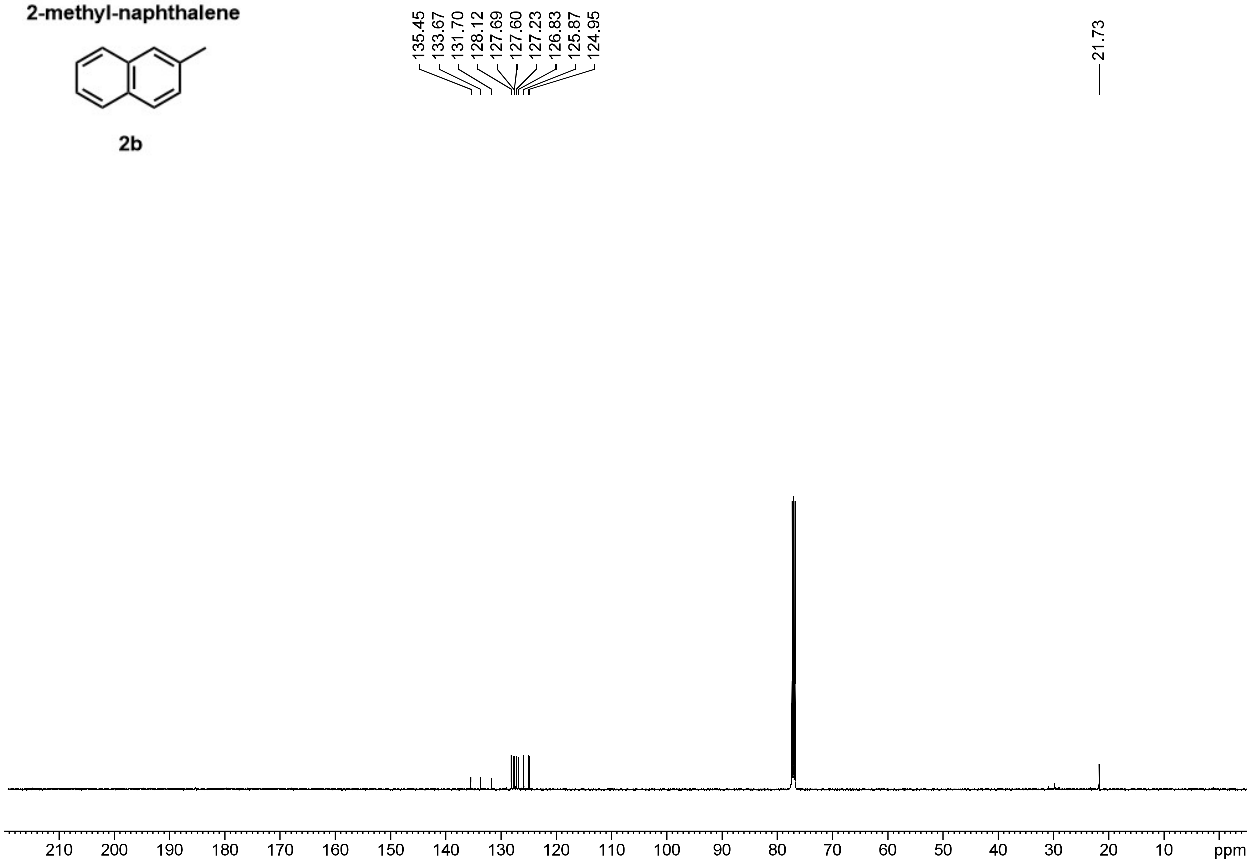
2-methyl-naphthalene
2b
135.45
133.67
131.70
128.12
127.69
127.60
127.23
126.83
125.87
124.95
21.73
210 200 190 180 170 160 150 140 130 120 110 100 90 80 70 60 50 40 30 20 10 ppm

1-methyl-naphthalene

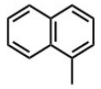

2c

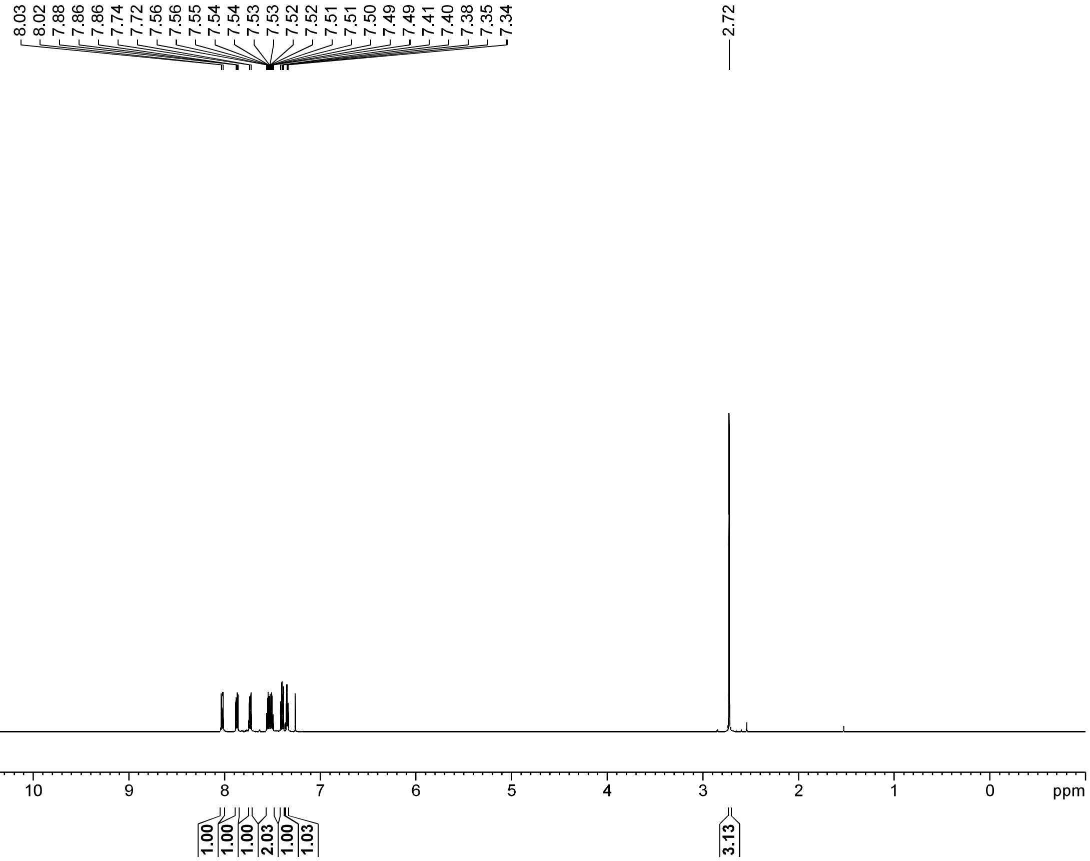

1-methyl-naphthalene

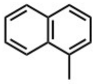

2c

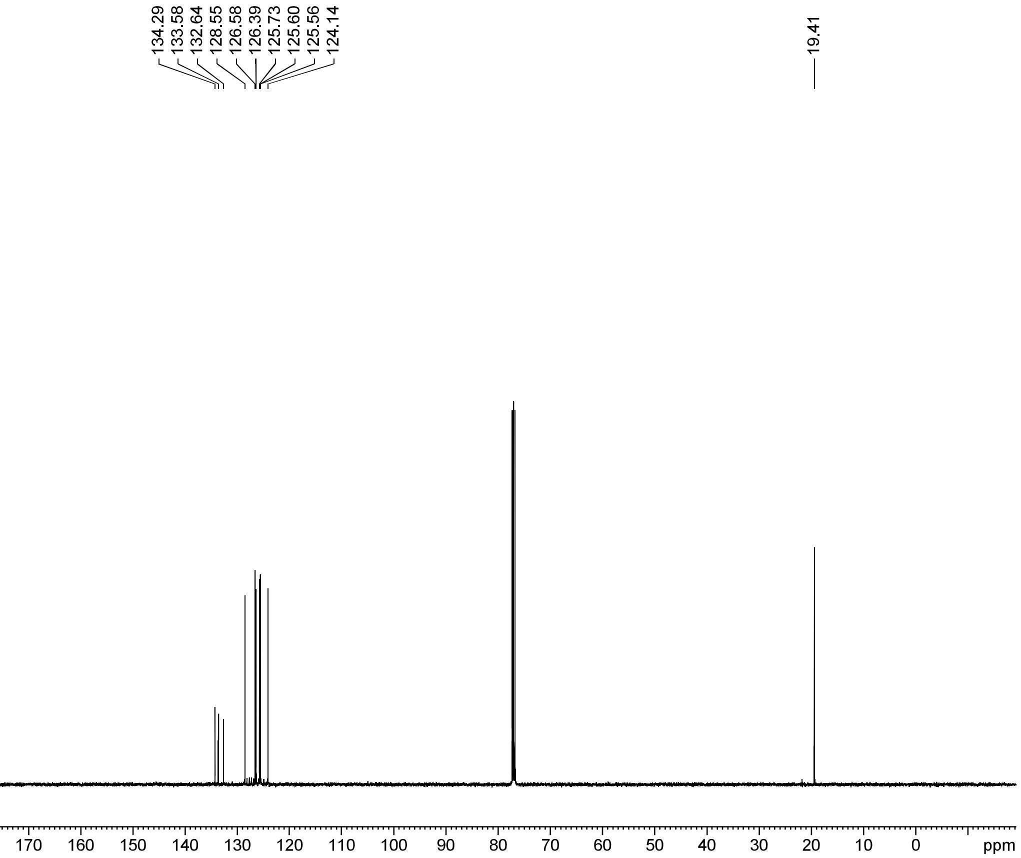

2-methoxy-naphthalene

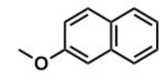

2d

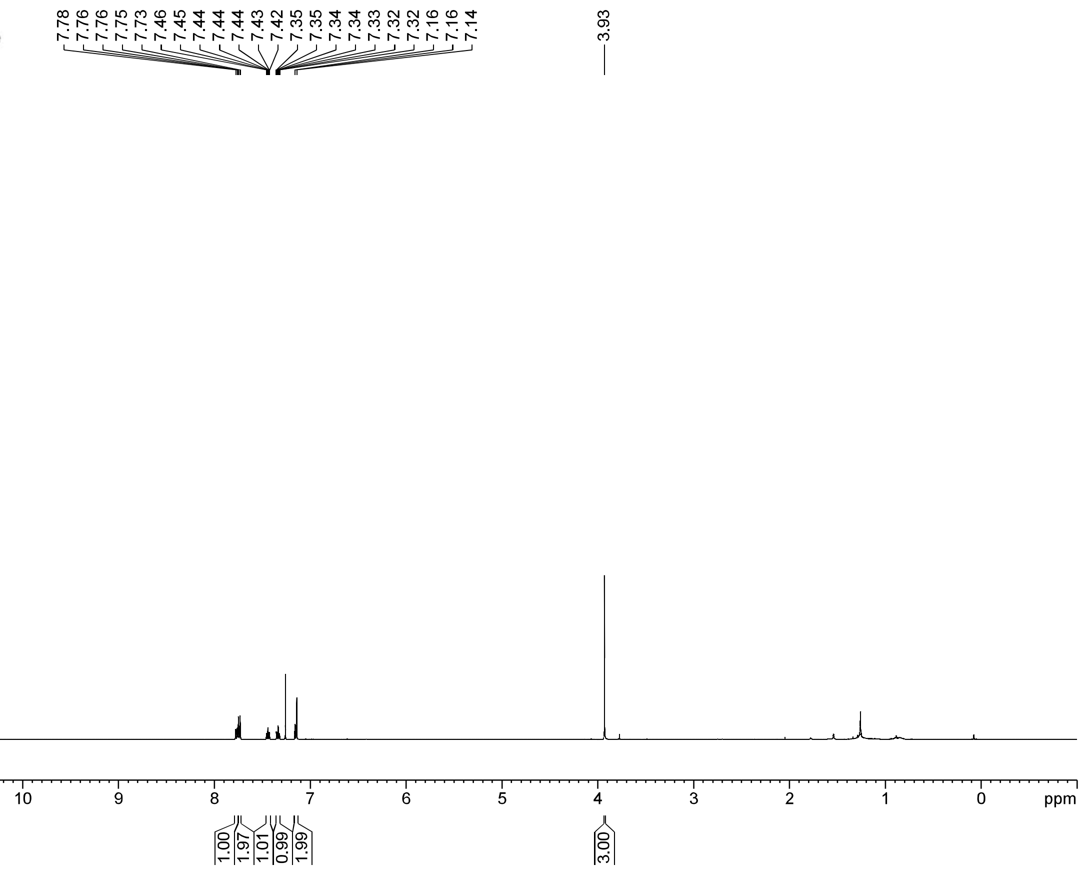

2-methoxy-naphthalene

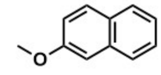

2d

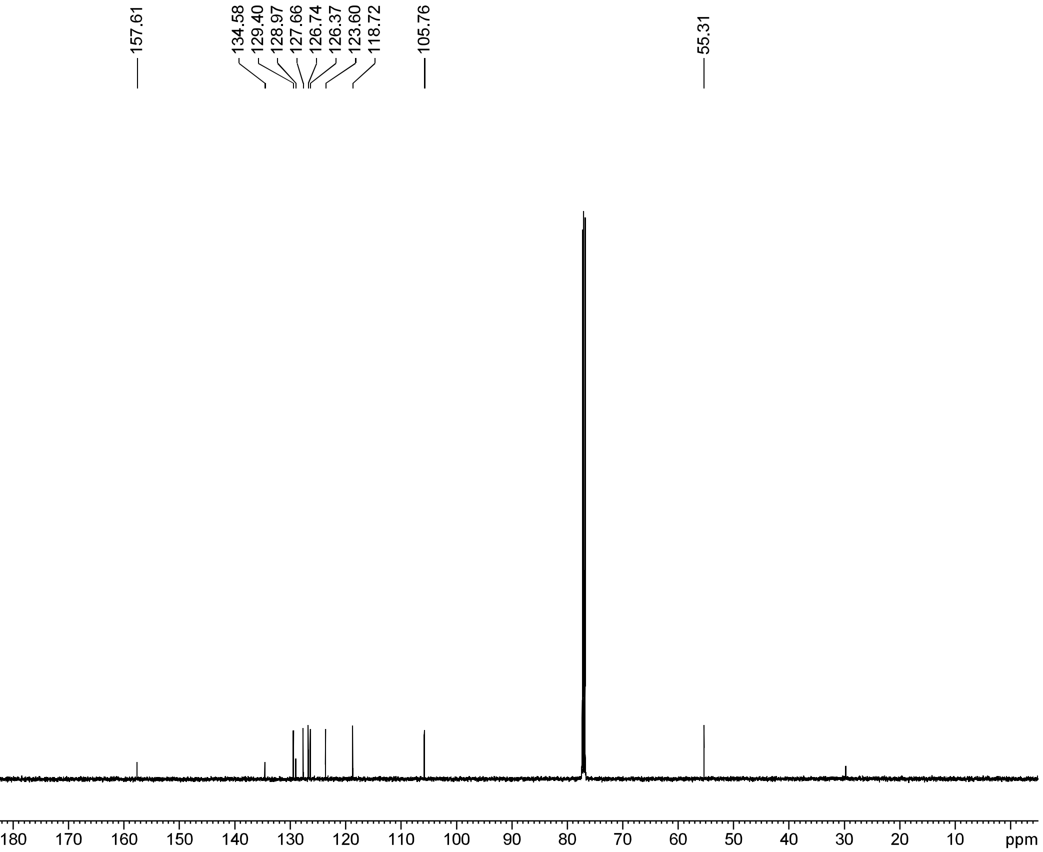

naphthalene

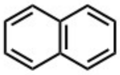

2a

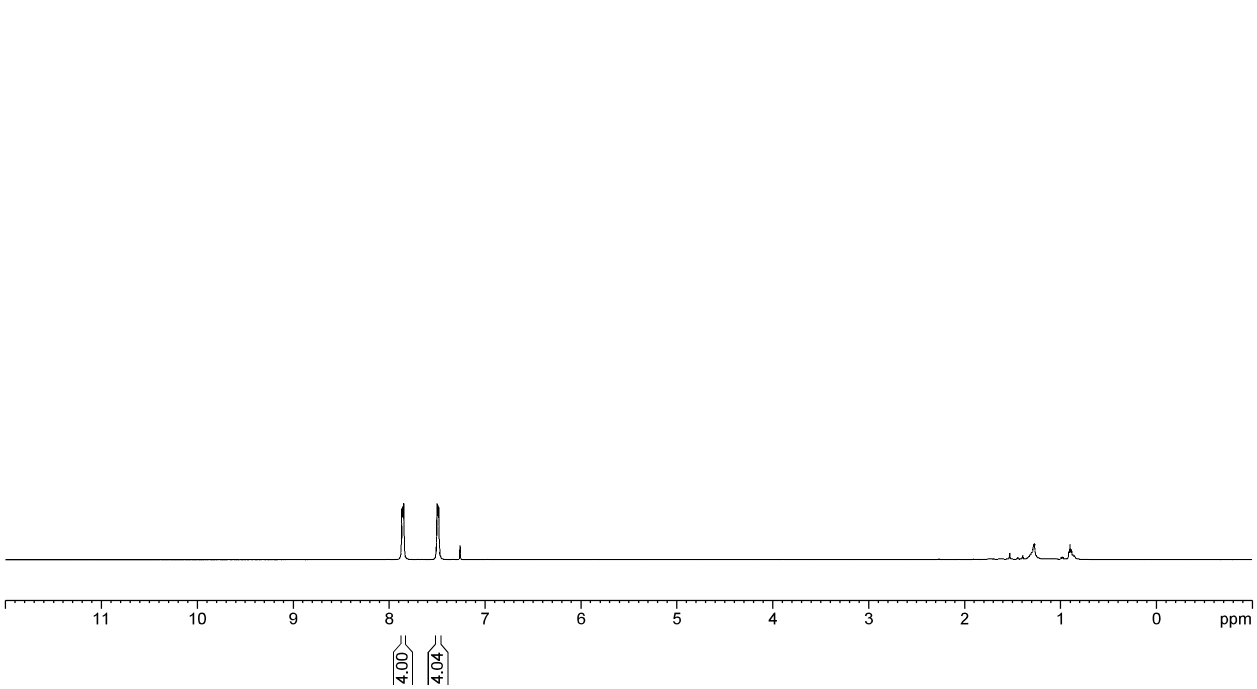

naphthalene

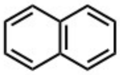

2a

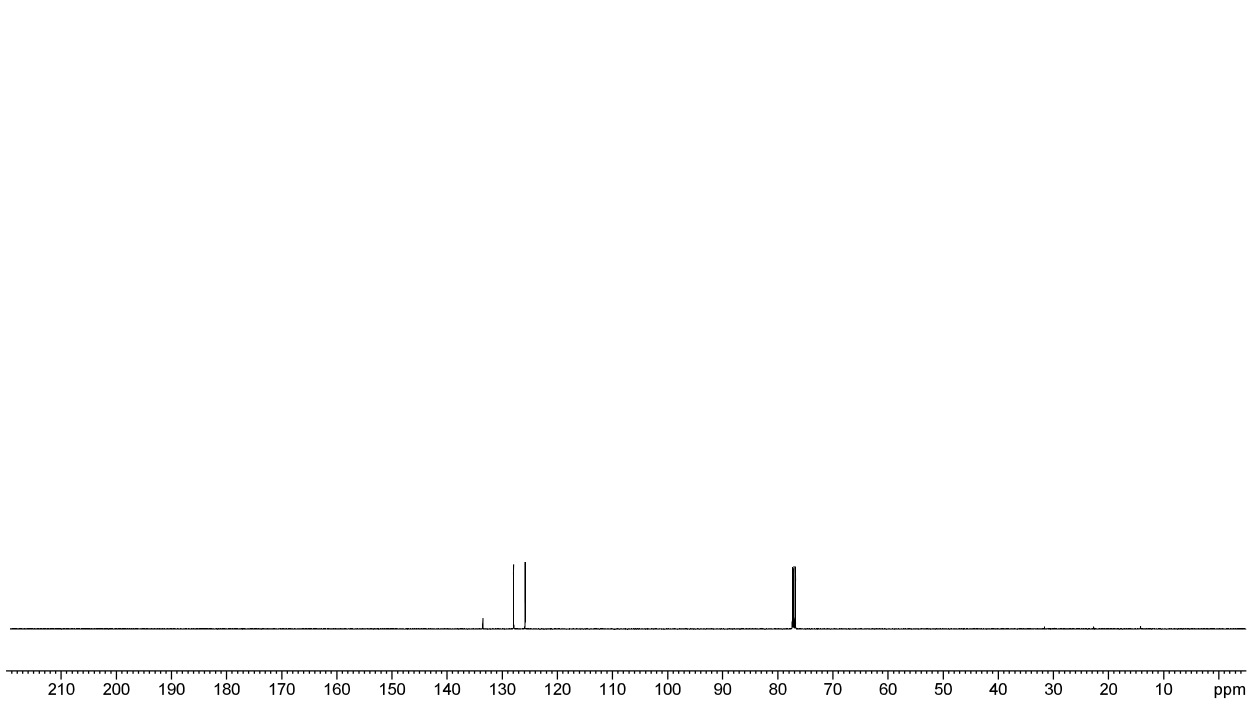

naphthalene

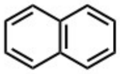

2a

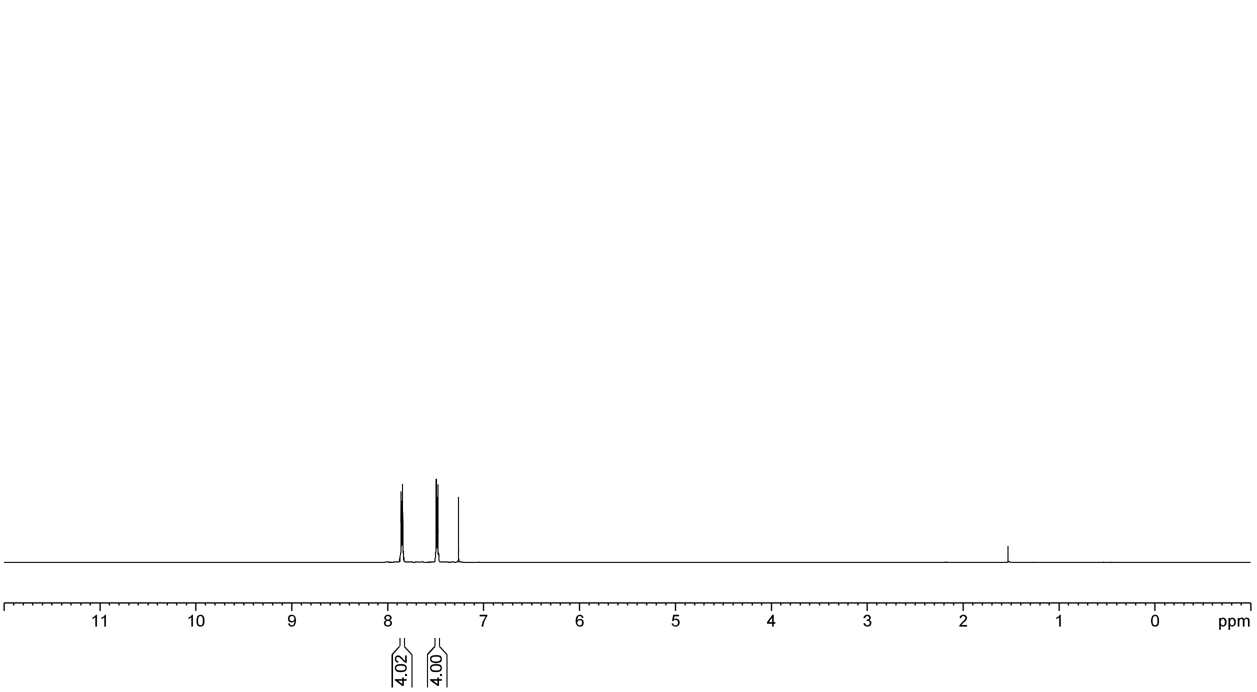

naphthalene

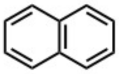

2a

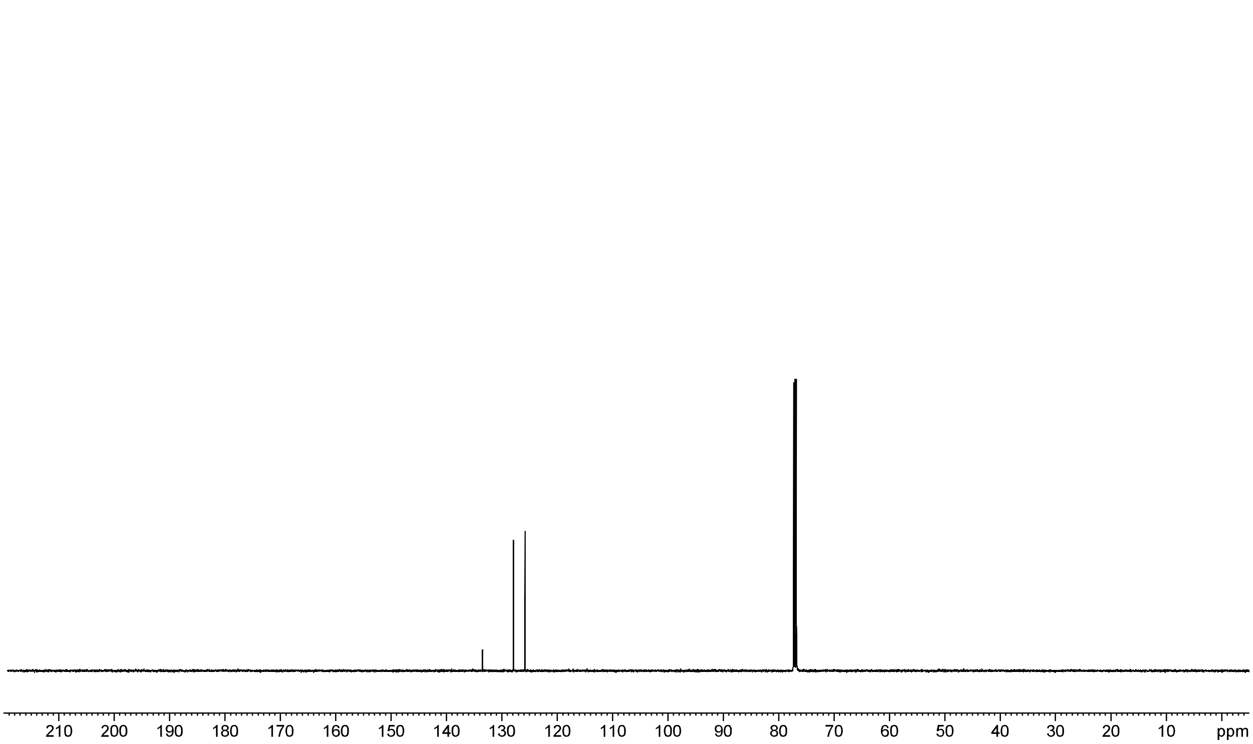

carbazole

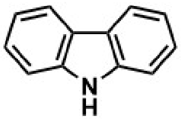

2g

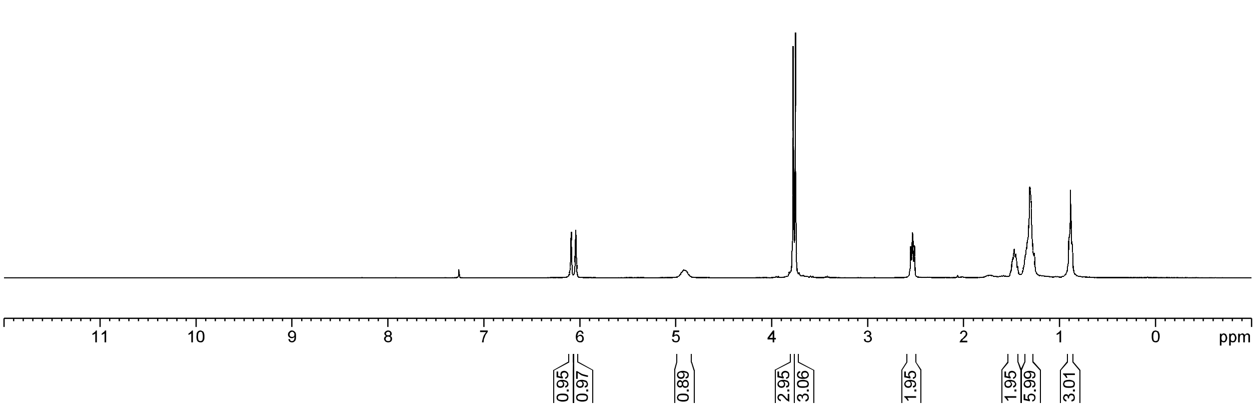

carbazole

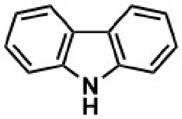

2g

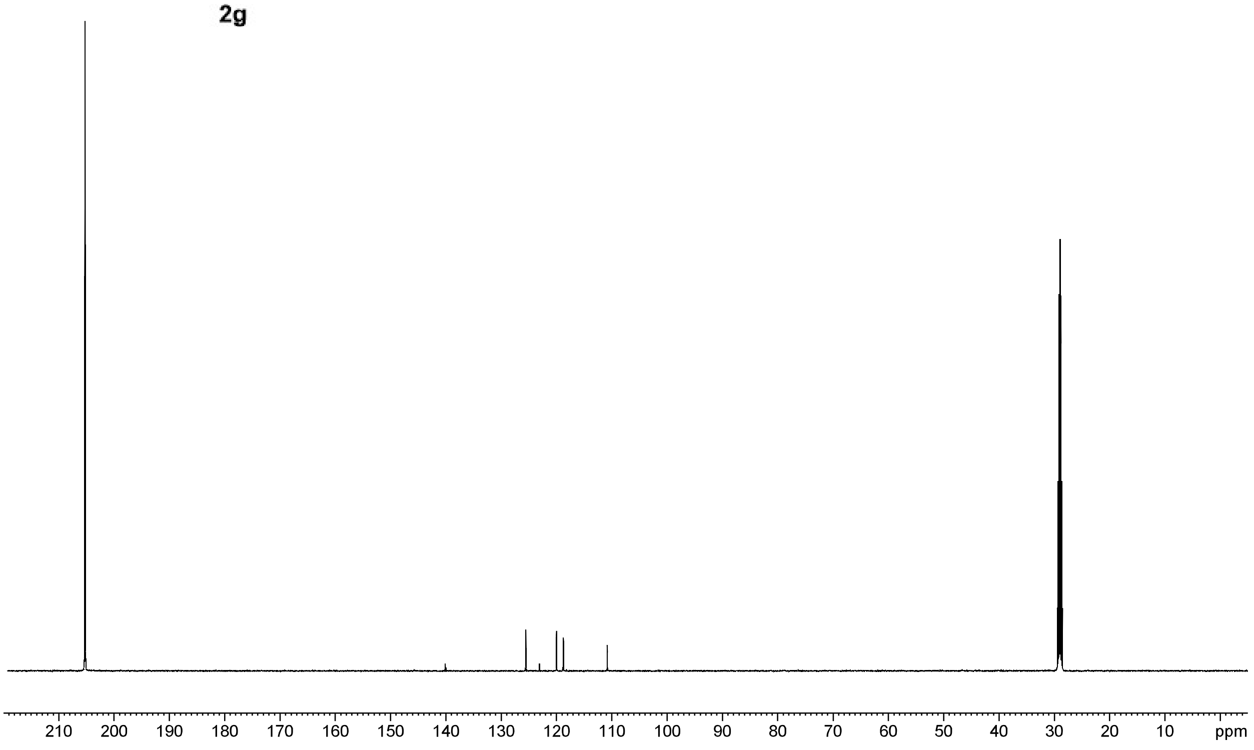

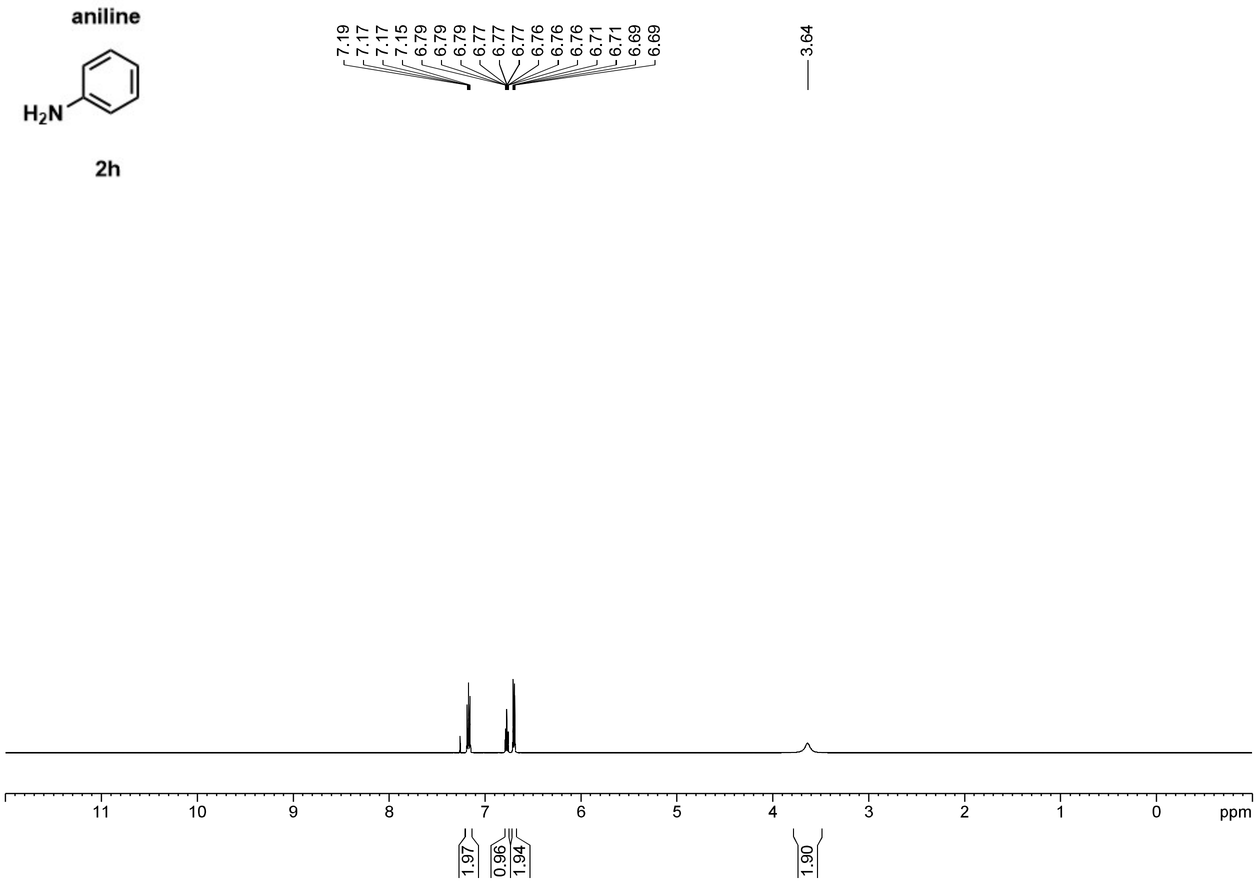
aniline
H_2N
2h
7.19 7.17 7.17 7.15 6.79 6.79 6.77 6.77 6.77 6.76 6.76 6.71 6.69 6.69
3.64
1.97 0.96 1.94 1.90
11 10 9 8 7 6 5 4 3 2 1 0 ppm

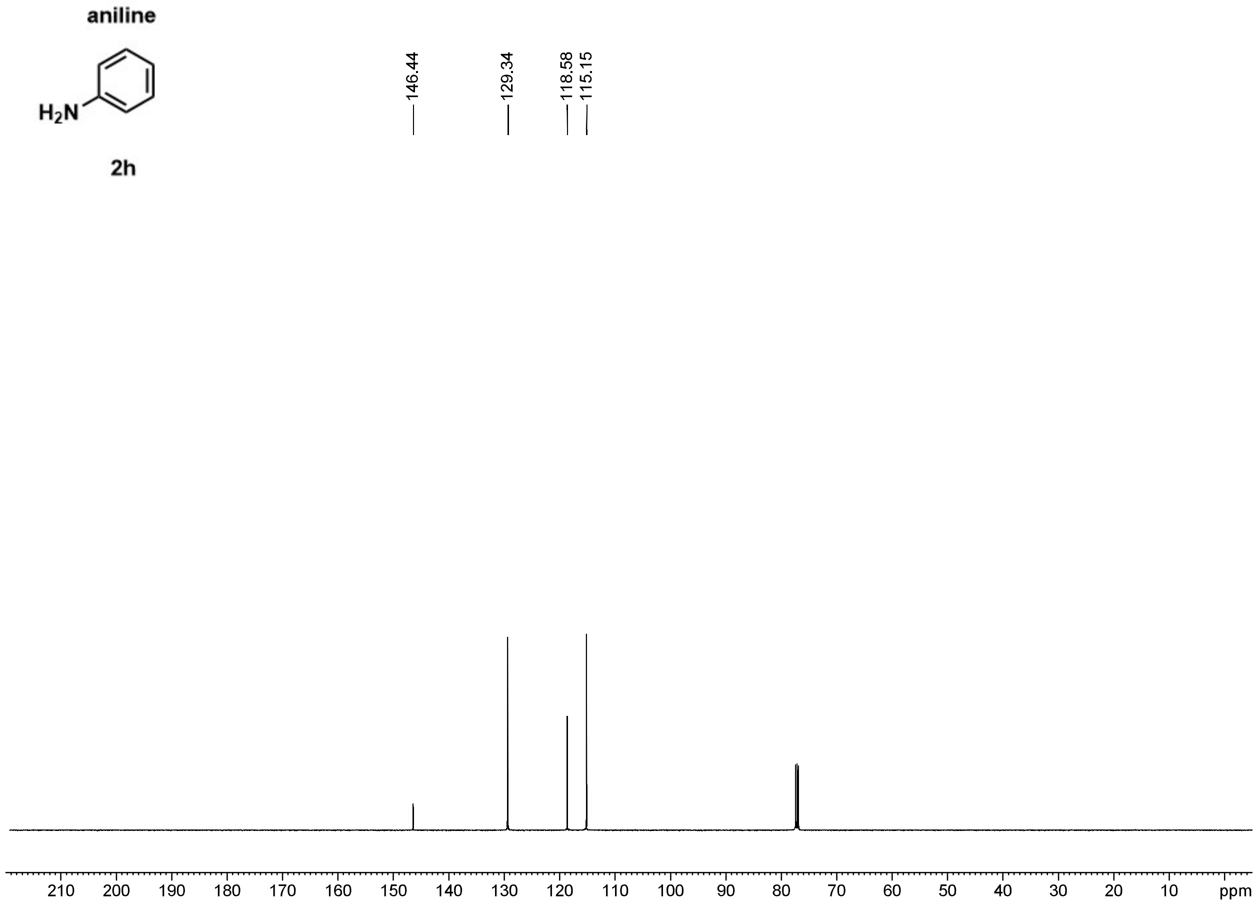
aniline
H_2N
2h
146.44
129.34
118.58 115.15
210 200 190 180 170 160 150 140 130 120 110 100 90 80 70 60 50 40 30 20 10 ppm

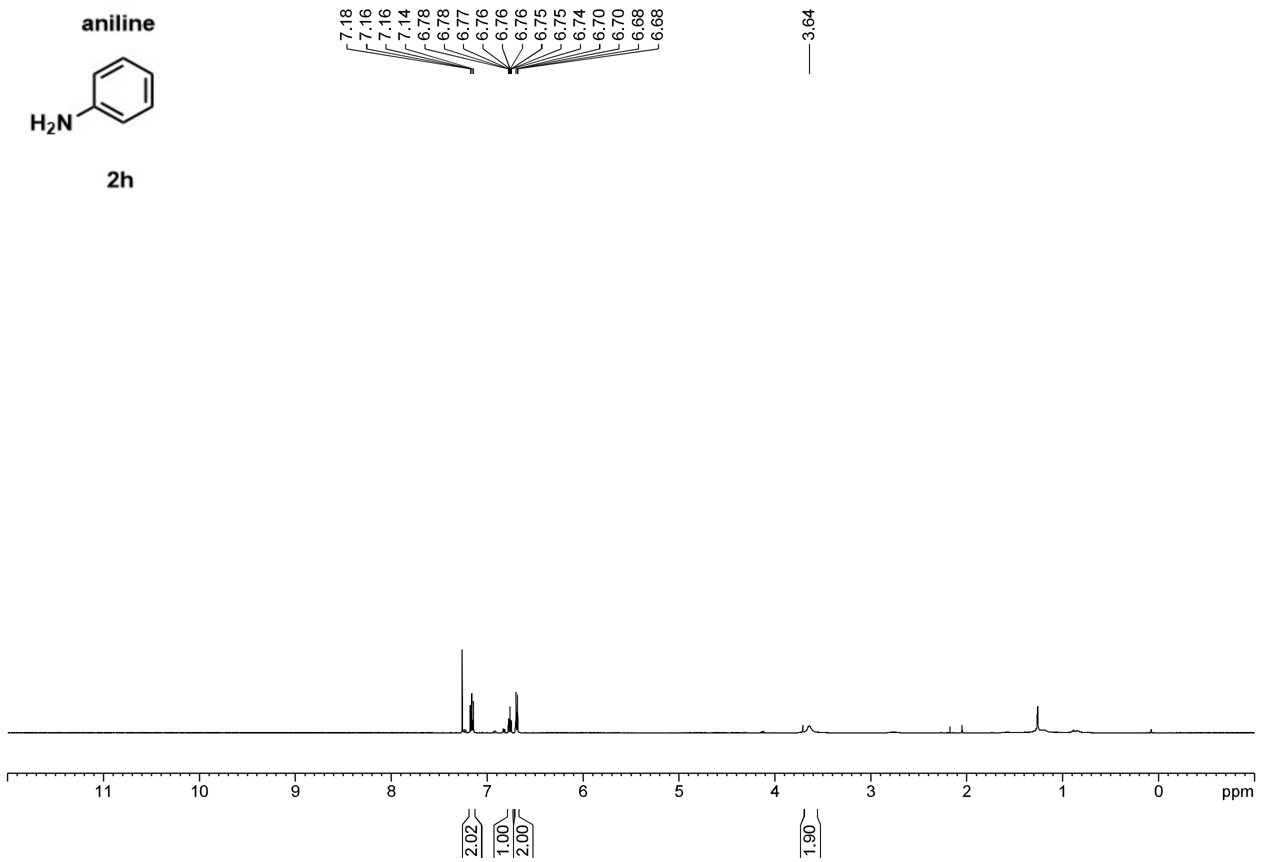

aniline
H_2N
2h
7.18 7.16 7.16 7.14 6.78 6.78 6.77 6.76 6.76 6.76 6.75 6.75 6.74 6.70 6.70 6.68 6.68
3.64
11 10 9 8 7 6 5 4 3 2 1 0 ppm
2.02 1.00 2.00
1.90

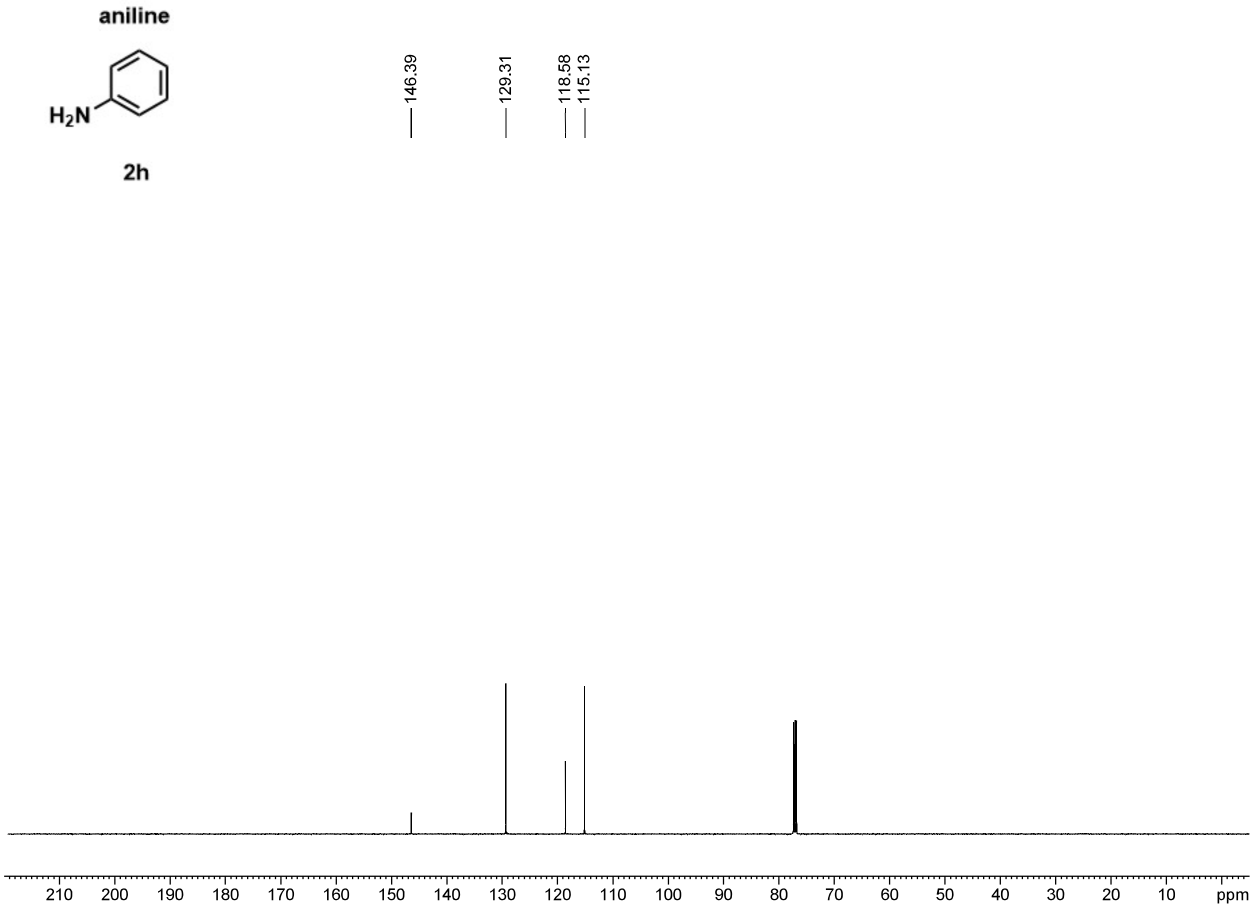

aniline
H_2N
2h
146.39
129.31
118.58 115.13
210 200 190 180 170 160 150 140 130 120 110 100 90 80 70 60 50 40 30 20 10 ppm

ethyl-benzene

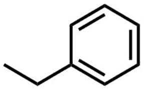

2j

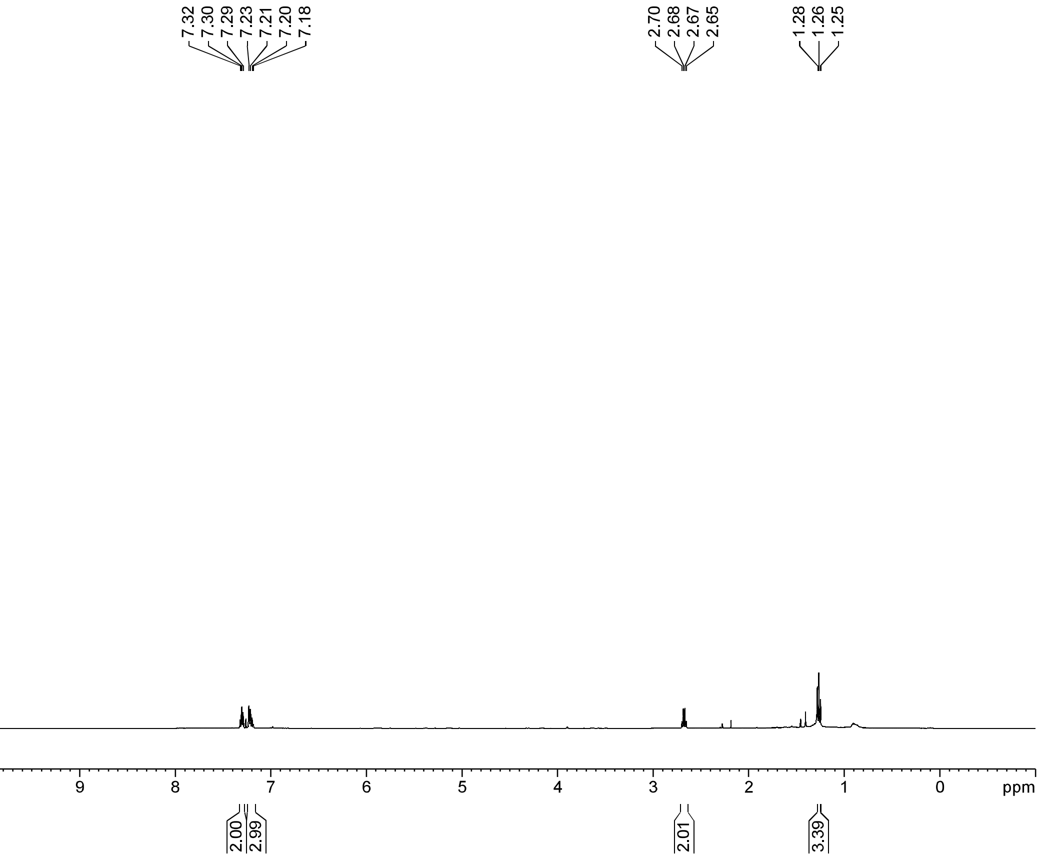

ethyl-benzene

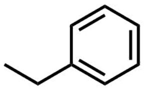

2j

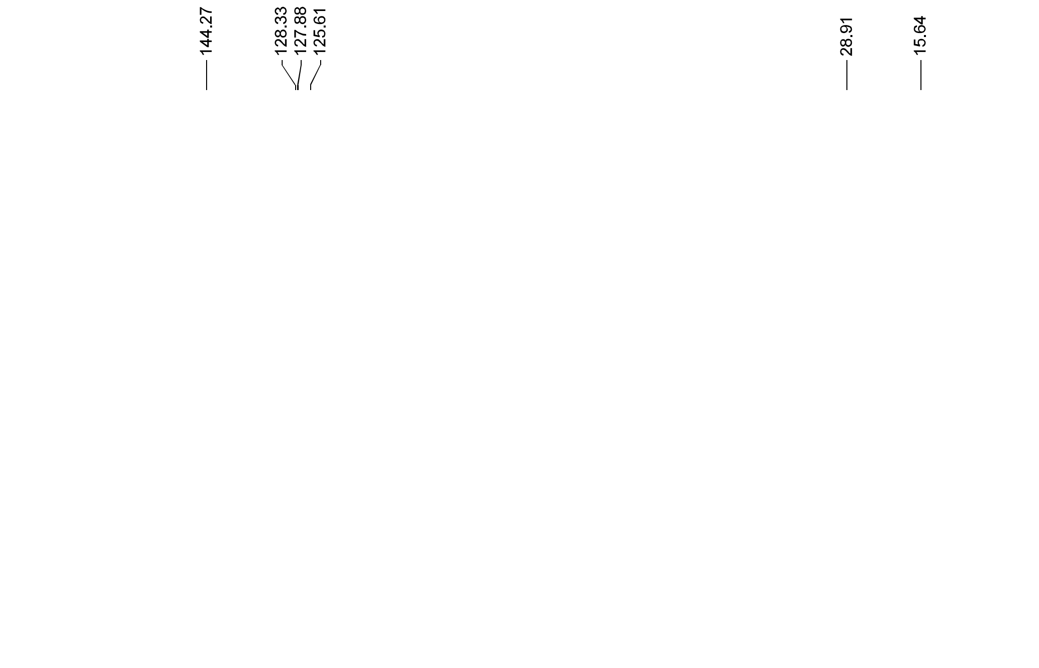

ethyl-benzene

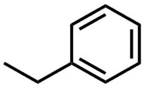

2j

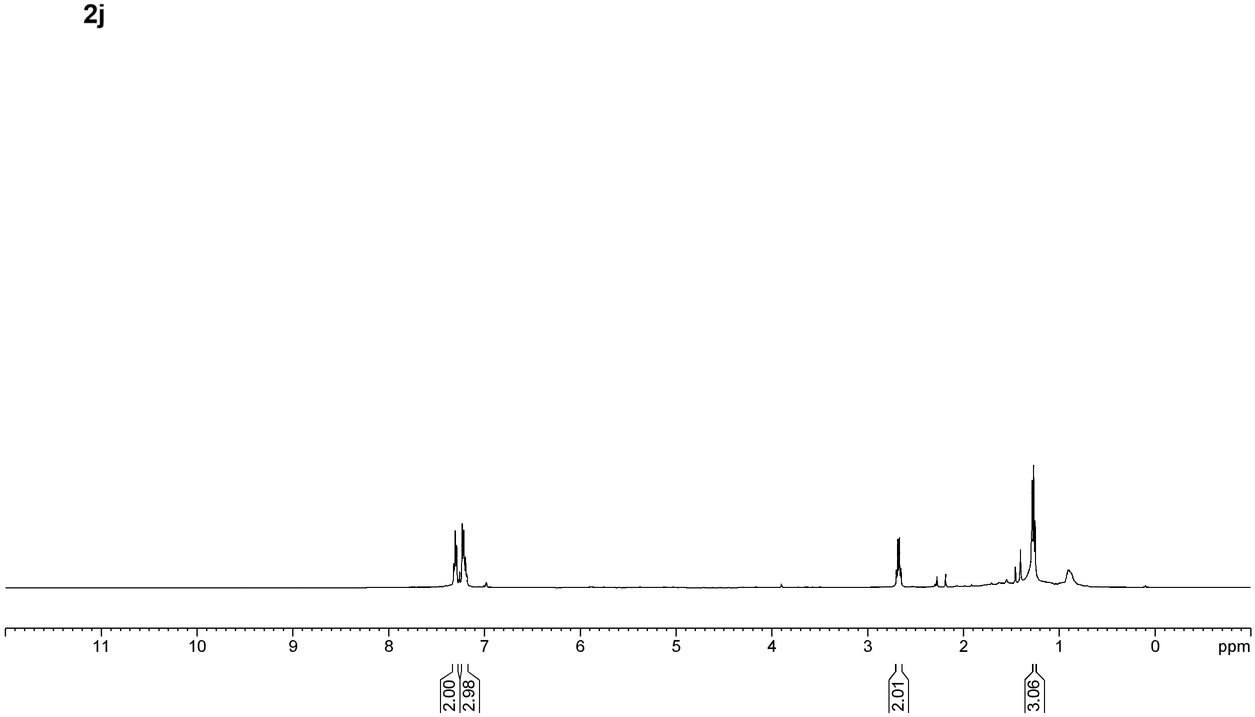

ethyl-benzene

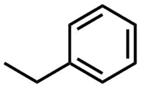

2j

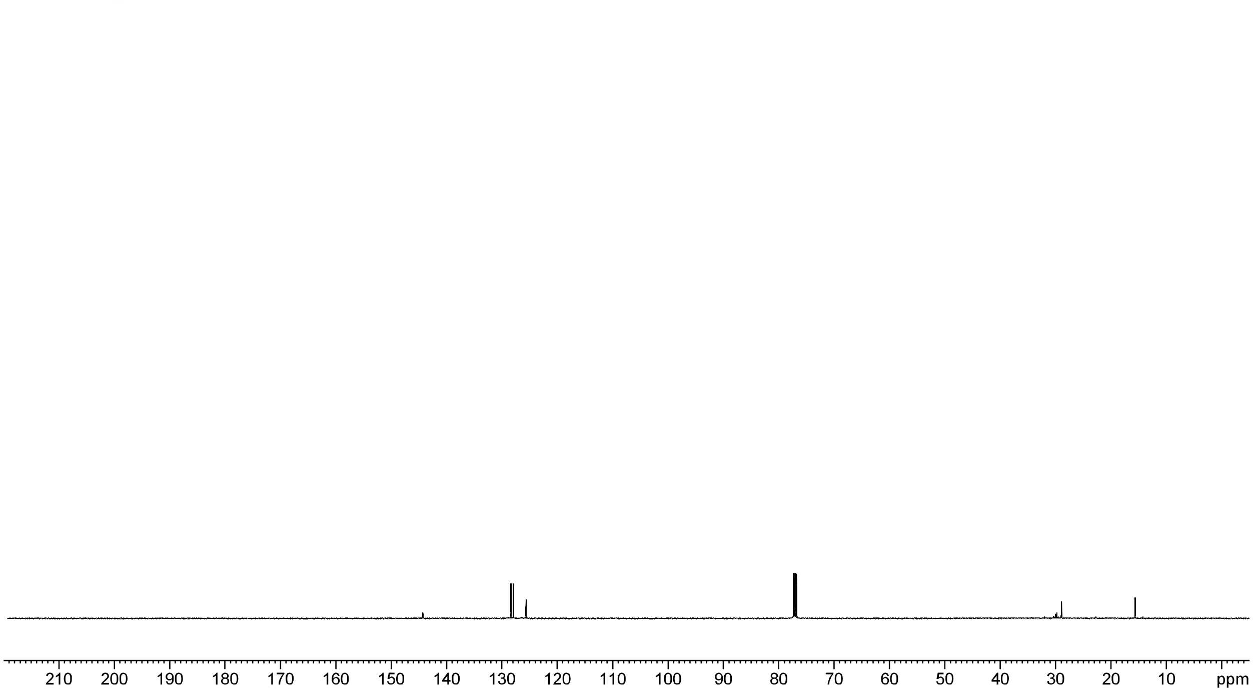

***p*-phenylphenol**

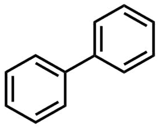

2l

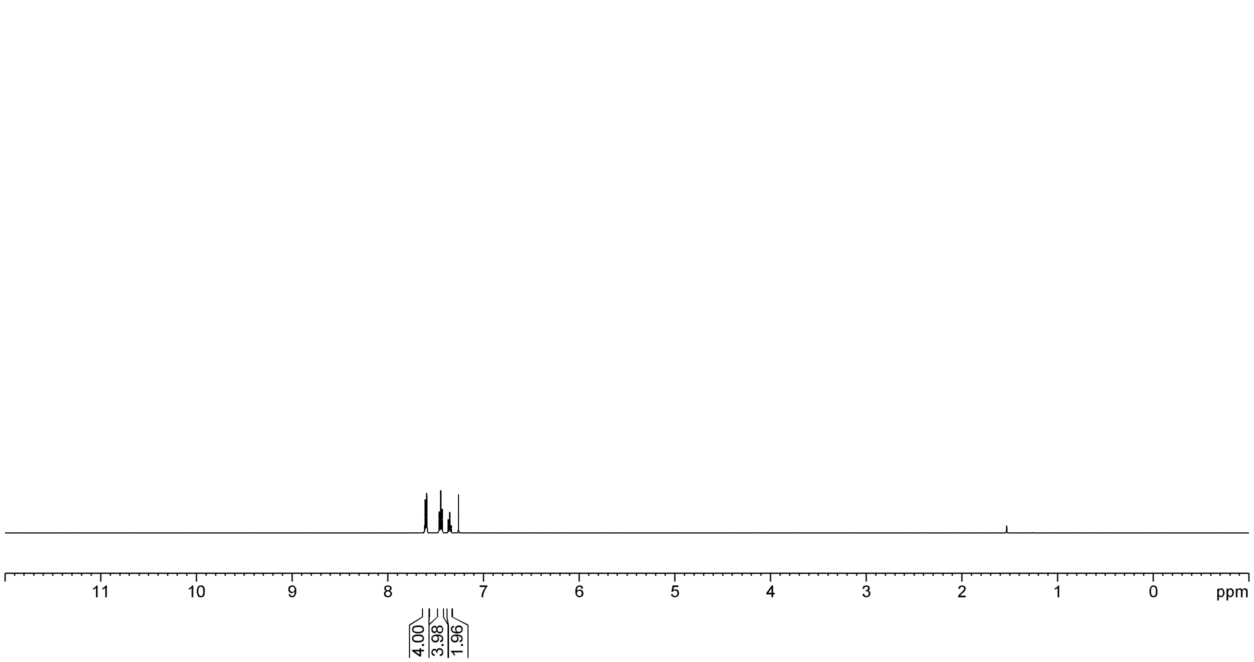

***p*-phenylphenol**

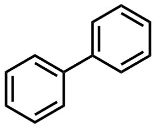

2l

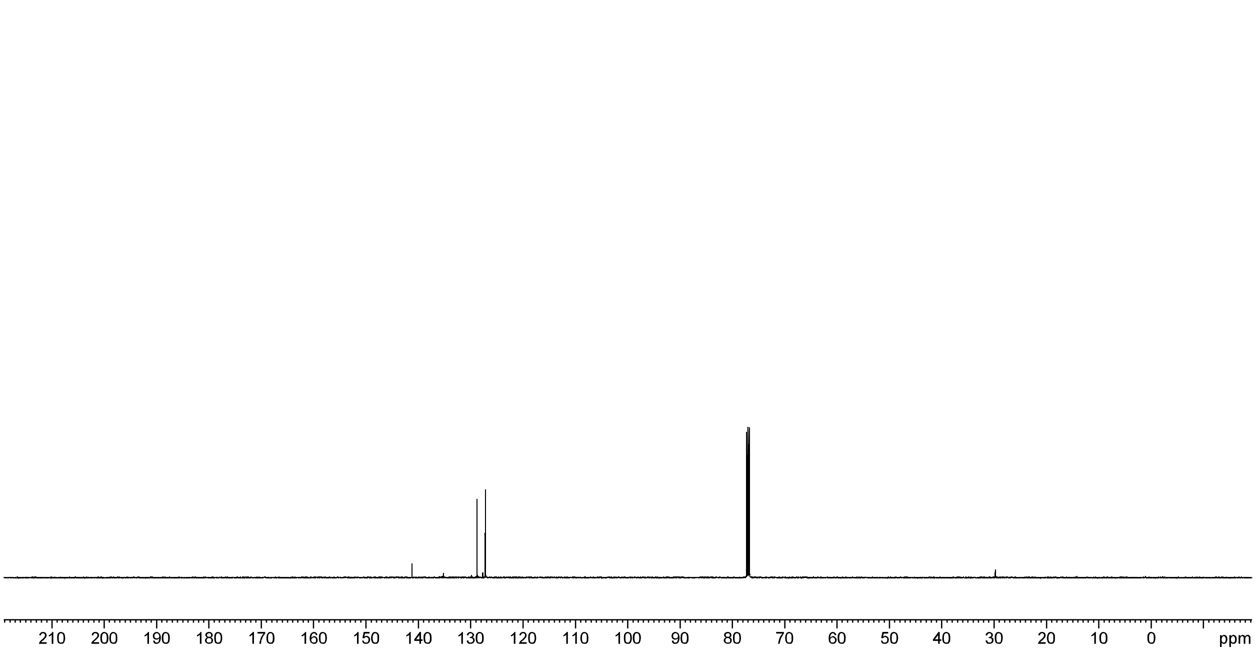

aniline

2h

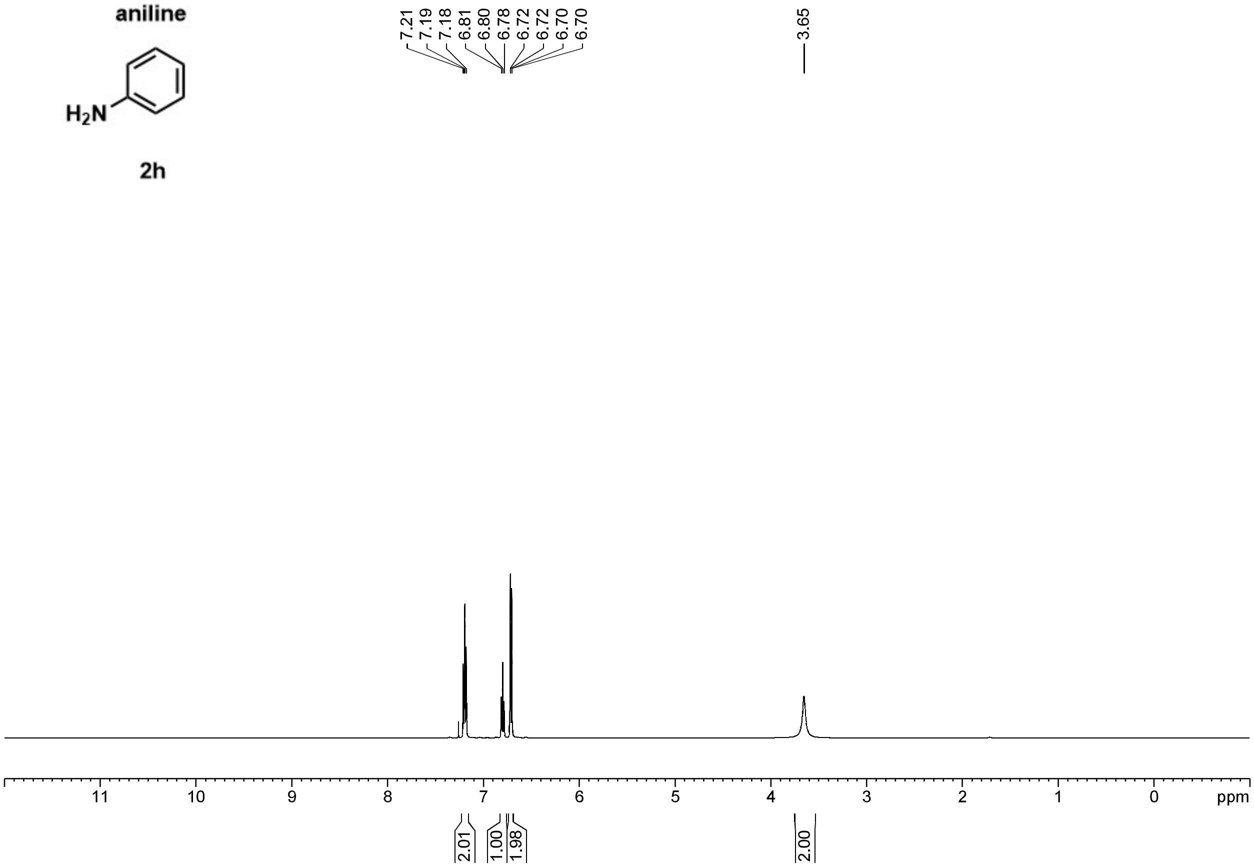

aniline

2h

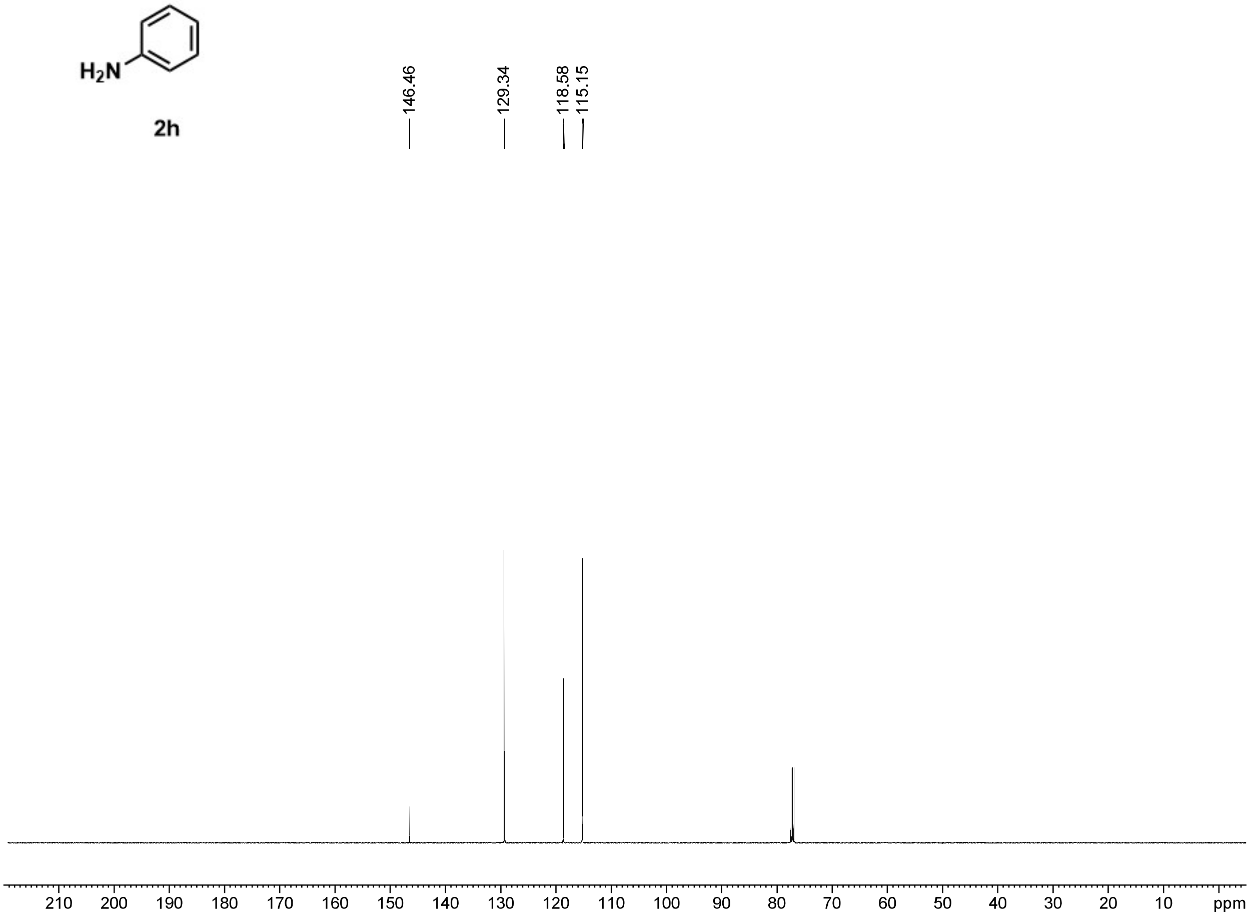

Supplement: Supplementary file 1 — cs4c06061_si_001.pdf [file cs4c06061_si_001.pdf]
